# Target-Derived Neurotrophic Factor Deprivation Puts Retinal Ganglion Cells on Death Row: Cold Hard Evidence and Caveats

**DOI:** 10.3390/ijms20174314

**Published:** 2019-09-03

**Authors:** Marie Claes, Lies De Groef, Lieve Moons

**Affiliations:** Laboratory of Neural Circuit Development and Regeneration, Department of Biology, KU Leuven, 3000 Leuven, Belgium

**Keywords:** retinal ganglion cells (RGCs), neurotrophic factors (NTFs), glaucoma, neuroprotection, optic neuropathies, optic nerve, axonal transport, retina, visual system

## Abstract

Glaucoma and other optic neuropathies are characterized by axonal transport deficits. Axonal cargo travels back and forth between the soma and the axon terminus, a mechanism ensuring homeostasis and the viability of a neuron. An example of vital molecules in the axonal cargo are neurotrophic factors (NTFs). Hindered retrograde transport can cause a scarcity of those factors in the retina, which in turn can tilt the fate of retinal ganglion cells (RGCs) towards apoptosis. This postulation is one of the most widely recognized theories to explain RGC death in the disease progression of glaucoma and is known as the NTF deprivation theory. For several decades, research has been focused on the use of NTFs as a novel neuroprotective glaucoma treatment. Until now, results in animal models have been promising, but translation to the clinic has been highly disappointing. Are we lacking important knowledge to lever NTF therapies towards the therapeutic armamentarium? Or did we get the wrong end of the stick regarding the NTF deprivation theory? In this review, we will tackle the existing evidence and caveats advocating for and against the target-derived NTF deprivation theory in glaucoma, whilst digging into associated therapy efforts.

## 1. Introduction

**Figure 1:**
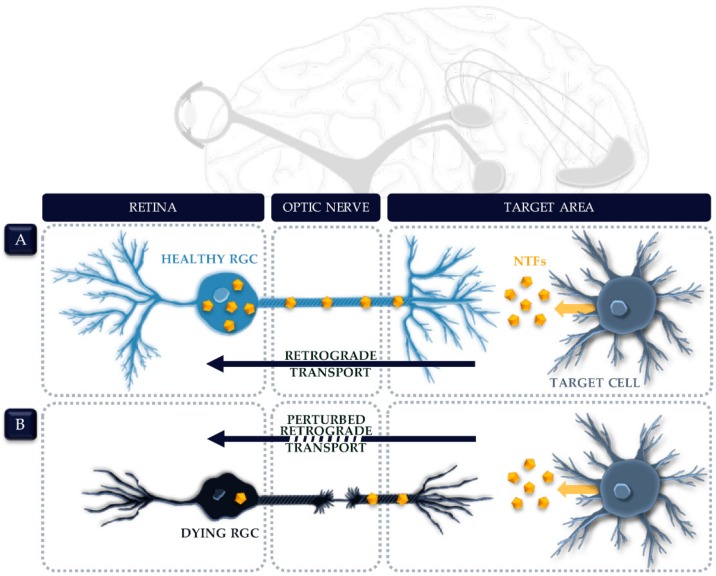
The neurotrophic factor (NTF) deprivation theory in the adult visual system. (**A**) Under physiological conditions, NTFs are secreted by cells in target brain centers and retrogradely transported alongside the optic nerve towards the retinal ganglion cells (RGCs). (**B**) In situations where axonal transport is perturbed—as witnessed in glaucoma and other optic neuropathies—RGCs are deprived from target-derived NTFs. According to the NTF deprivation theory, this scarcity is the final push that forces the struggling RGCs to surrender to the injury stressors and undergo apoptosis.

A common theme in the nervous system is the two-way axonal stream between neurons and their target areas. To ensure the homeostasis and viability of the innervating neuron, essential molecules are transported along this axonal highway from the cell soma to the axon terminus—i.e., anterograde transport—and the other way around—i.e., retrograde transport. Retinal ganglion cells (RGCs), positioned in the inner retina, form no exception to this. Their axons collectively bundle into the optic nerve, which guides visual information towards target brain centers. As such, the optic nerve shapes the vital connection between the eye and the brain. Consequences of a disruption or any other damage to this linkage—as witnessed in optic neuropathies—cannot be underestimated. The most common optic neuropathy is glaucoma, in which progressive degeneration of RGCs and their axons gradually culminates into irreversible blindness [1]. Despite the extensive research performed to unravel the pathogenesis of glaucoma, the initial event that triggers the start of RGC degeneration is yet unknown [2]. Nowadays, glaucoma is viewed as a complex multifactorial disease, referring to the multitude of possible underlying pathological mechanisms. The identity, sequence, as well as the interplay between various contributing factors is still highly debated [3,4]. One piece of the glaucoma puzzle is the theory around target-derived deprivation of neurotrophic factors (NTFs) [3,5,6,7,8]. This theory arose in the glaucoma research field from the observation that failure of axonal transport is an early hallmark, presumably proceeding cell soma degeneration [9,10]. Hindered retrograde transport might cause a deficiency of crucial, target-derived survival factors at the cell soma [11]. One class of such important factors traveling retrogradely alongside the axonal highway are NTFs. These diffusible peptides regulate neuronal survival in the developing, adult and injured/diseased nervous system [12]. Regardless of the instigating mechanism of axonal transport malfunction—e.g., physical compression, reduced blood supply, and energy deficiency, etc.—the NTF deprivation theory states that the scarcity of those vital target-derived NTFs triggers RGC death (Figure 1) [3,5,6,7,8,9,13].

The NTF deprivation theory is not confined to glaucoma and might also apply to other optic neuropathies—e.g., optic neuritis and Leber hereditary optic neuropathy—and even to other neurodegenerative diseases in which axonal transport and retinal function is compromised—e.g., Parkinson’s and Alzheimer’s diseases [14]. The broad variety of diseases in which NTF deprivation might be involved denotes that it is highly unlikely that NTF deprivation is the initial trigger causing neuronal degeneration. Nowadays, NTFs are reckoned as generic mediators of neuronal survival in the naive, aging and injured/diseased nervous system. When experiencing stress, the destiny of a neuron balances between survival and apoptosis. NTFs represent a counterbalance towards neuronal survival as they are known to increase the stress resistance of a neuron. Absence of this vital NTF support will send the neurons unarmed to the battle field and almost destine them to surrender. Of note, NTFs function independently of the kind of stress that makes the neuron prone to die. As such, NTF supplementation has been glorified as a potential treatment for various neurodegenerative diseases—such as age-related macular degeneration, amyotrophic lateral sclerosis, Parkinson’s disease, Alzheimer’s disease, etc. [15,16]. In glaucoma, NTF supplementation showed auspicious results in animal models, yet clinical translation keeps failing as NTF therapy shows minimal or no effects in patients. In order to lever NTF therapies towards the therapeutic armamentarium, we might need to reevaluate the target-derived NTF deprivation theory.

## 2. Which NTFs and Signaling Pathways Are Involved?

Although there is often a mixed usage of the terms “neurotrophic factors” and “neurotrophins”, they are not interchangeable. NTFs is a collective name for all secreted neuron-supporting peptides, which can be broadly classified into four superfamilies: the neurotrophin, neurokine/neuropoetin, transforming growth factor β (TGF-β) and non-neuronal factor superfamilies [17,18]. Amongst all, the neurotrophins are appointed as the most potent survival agents in the central nervous system, and thus received the most attention in glaucoma research. The classical neurotrophin family consists out of four well-defined members: nerve growth factor (NGF), brain-derived neurotrophic factor (BDNF), neurotrophin-3 (NT-3) and neurotrophin-4/5 (NT-4/5). In contrast to mammals, two additional neurotrophins have been identified in fish: neurotrophin-6 (NT-6) and neurotrophin-7 (NT-7) [19,20]. NT-6 and -7 are most closely related to NGF, but mammalian orthologs have not been found [21]. Besides these neurotrophins, ciliary nerve trophic factor (CNTF) and glial cell-derived neurotrophic factor (GDNF) are often cited as potent neuroprotective agents in glaucoma. They belong to the neurokine/neuropoetin and TGF-β superfamily respectively. Although NTF deprivation is not considered as the primary cause of glaucoma, evidence for a linkage between NTFs and two of the most prevalent glaucoma risk genes—myocilin and optineurin—has been found. For example, NGF treatment increased the endogenous levels of both genes in PC12 cells [22] and a deficiency of optineurin was shown to reduce the secretion of NT-3 and CNTF in vitro [23]. On the other hand, genome-wide association studies show a correlation between mutations in some NTF genes—NT-4 and BDNF—and the occurrence of glaucoma [24,25,26]. However, NTF mutations alone are almost certainly insufficient to trigger the onset of glaucomatous damage, as multiple studies have shown that these genetic mutations are very heterogenous and clearly vary with different ethnicities [27,28]. 

Literature screening revealed a striking dispersion of studies investigating NTFs in the naive and injured visual system and clearly denotes a lack of comprehensive studies. Moreover, researchers seem to be wearing blinders when studying NTFs as they mostly focus on the abovementioned NTFs, of which the survival promoting functions are already validated in the (visual) nervous system. Nevertheless, other NTFs or molecules not yet appointed to the NTF family could potentially be as powerful or even more promising neuroprotectants. Up till now, an unbiased screening to reveal the complete spectrum of retrogradely transported NTFs in the optic tract is missing and as such, the exact identity of each NTF in the vital cocktail for RGC survival remains elusive.

NTFs exert their function by binding to cell surface receptors and consequently triggering various intracellular signaling pathways involved in neuronal survival [29,30]. The best studied NTF pathways include the mitogen-activated protein kinase/extracellular-signal-regulated kinase (MAPK/ERK), phospholipase C-γ (PLC-γ), phosphoinositol-3 kinase/protein kinase B (PI3K/AKT), Janus kinase/signal transducers and activators of transcription (JAK/STAT) and nuclear factor kappa-light-chain-enhancer of activated B cells (NF-κB) pathways [31]. On the other hand, NTFs are also associated with the activation of the pro-apoptotic c-Jun N-terminal kinase (JNK) cascade [30,32,33,34,35,36]. This labyrinth of divers signaling pathways and distinct biological outcomes, already hints at the complexity of NTF functioning. Many variations exist along each step of the NTF action mechanism, as multiple NTFs, receptors, associated signaling molecules, downstream targets, variants of signaling pathways, etc. are involved, which most probably intersect and crosstalk to influence the specific biological outcome. Although they were first identified as proteins affecting cell fate, NTFs are now known to be involved in a broad variety of other cellular responses like proliferation, differentiation, neurite outgrowth/remodeling, cytoskeleton remodeling, membrane vesicle transport, synaptic functions, etc. [37]. It is therefore highly likely that the biological outcome will hinge on the specific cell type and settings. This notion is of particular importance since the existence and functioning of NTFs is not limited to the visual system but observed throughout the entire nervous system. Moreover, the vast majority of our current knowledge regarding NTF signaling is derived from in vitro studies, which often exclude the specific microenvironment of a certain tissue. Commonly used cultured cells in NTF research are PC12 cells, which come with the major drawback of having a limited NTF receptor expression [20,37]. Hence, only the signaling pathways of a small number of NTFs can be interrogated. Most of these studies have been focusing on NGF, the first identified and best characterized NTF. Unfortunately, extrapolating NGF signaling pathways to other neurotrophins or NTFs is not straightforward. For example, although NGF and NT-3 bind the same receptor, they do mediate a divers output [38]. While it is recognized that each NTF most probably induces a (slightly) different set of downstream actuators, the complete cobweb of molecular players for every particular NTF is not yet uncovered and might even differ in distinct tissues. Nevertheless, signaling pathways previously linked to NTF actions in PC12 cells have often been shown to be up- or downregulated in animal glaucoma models, as reviewed by Levkovitch-Verbin [31]. An important note is that the vast majority of these reports select one or more downstream actuators of a specific signaling pathway and examine their phosphorylation state in a glaucoma setting via Western blot. Hence, the exact identity and in vivo mechanism of action of NTFs in the visual system is not completely resolved. We believe that all of the abovementioned gaps in the current NTF knowledge are striking and need to be addressed prior to continuing the search towards therapeutic applications for NTFs.

## 3. NTFs in the Developing and Adult Nervous System

Before digging into the current knowledge upholding the NTF deprivation theory, we first wish to tackle the assumption that target-derived NTFs exert a similar role in the survival of adult neurons as in developing neurons. During development, an overproduction of neurons ensures fine-tuning of proper target innervation: neurons that fail to adequately connect to their target—and NTF source—area, undergo programmed cell death [29,39,40,41,42,43,44]. Experimental results document conclusively that this is governed by the competition for limited quantities of target-derived NTFs. Hence, the acquired level of target-derived NTFs tilts the fate of the neuron towards survival or death [30]. Of note, cell death regulation during development by means of NTF competition is referred to as the “neurotrophic theory”, not to be confused with the NTF deprivation theory reviewed here [29,45,46]. Retinal axons projects to more than 50 target areas, of which the dorsal lateral geniculate nucleus (dLGN) and the superior colliculus (SC)—or its nonmammalian homolog, the optic tectum—are the most prominent, depending on the species [47,48].

Target-derived NTFs clearly play a key role in the survival of developing neurons and cited evidence for the NTF deprivation theory in the adult injured nervous system often builds on these observations. However, the dependency of NTF support for neuronal survival can differ over the course of the lifetime of a neuron. After proper target innervation, the necessary NTF support for neuronal survival might shift from a target-derived to a local mode of action [49]. This hypothesis is supported by discordant observations after lesion or removal of the major RGC target structure in neonatal and adult animals. Ablation of the SC or dLGN in neonatal rodents caused an acute and substantial loss of RGCs [50,51,52]. Per contra, after SC ablation in adult rodents, significant RGC loss was not observed [53] and destruction of the dLGN in adult cats only caused RGC death several months later [54]. This postponed RGC death indeed points out a reduced dependency of RGCs on functional target areas. It should however be noted that mature RGCs can project to multiple target areas [55] and lesion/ablation of a sole target structure does not imply full deprivation of target-derived support. An interesting finding is that target ablation or axonal transport blockage—either by lidocaine or by optic nerve injury (ONI)—in adult rodents does result in a rapid reduction of RGC functionality [53,56]. Of note, this finding is often overlooked in current NTF studies. The majority of them report RGC survival, but neglect RGC functionality, whilst being highly important for future clinical approaches. To conclude, functioning of mature RGCs probably continuous to depend on intact target areas and/or retrograde axonal transport, albeit to another degree as compared to neonatal RGCs. When drawing a conclusion about NTF support, one should always disconnect studies that examine the developing and mature nervous system as target-derived NTF dependency might differ in both settings. The following sections will primarily focus on studies in the adult nervous system, unless indicated otherwise.

## 4. Which Tangible Evidence Corroborates the NTF Deprivation Theory in Glaucoma?

### 4.1. Axonal Transport Deficits in Glaucoma

It has been shown that purposefully blocking axonal transport by ONI or lidocaine application rapidly leads to a decline in RGC functionality and eventually culminates into cell death [57]. Even in animal glaucoma models without direct interference with axonal transport—such as the experimental laser photocoagulation or vein cauterization model—deficits in axonal transport were reported. This phenomenon was mainly studied by applying neurotracers like FluoroGold to target centers in the brain and assessing the retrograde labeling of RGCs [58,59,60,61,62,63,64] or by exploring axonal cytoskeletal/organelles abnormalities—such as the accumulation of dynein motor proteins, mitochondria and microvesicles [65,66]. Axonal transport deficits have also been documented in the DBA/2J mouse, which spontaneously develops glaucoma with age [67,68]. Likewise, post mortem studies of eyes of human glaucoma patients revealed axonal abnormalities [9] and local disruption of axonal transport [69]. Besides retrograde failure, also anterograde transport is affected in glaucoma models [61,62,70,71,72,73]. At first sight, this might seem irrelevant for the NTF factor deprivation theory, but one should bear in mind that NTF receptors are assembled in the RGC soma and need to be anterogradely transported towards the axon termini to ensure continued responses to target-derived NTFs [74,75]. As such, both failure of anterograde and retrograde transport espouses the NTF deprivation theory. Of note, hindrance of axonal transport is assumed to precede RGC death [63,64,76]. The latter is an important notice since it proves that axonal transport discrepancies are not a result of RGC death, but rather contribute to it by inducing and/or amplifying cell death in glaucomatous settings. Hence, well-functioning axonal transport seems to be a prerequisite for RGC functionality and survival.

### 4.2. NTF Transport along the Optic Nerve

The next question that arises is: what proof do we have that NTFs are transported along the optic nerve? First of all, the aforementioned NTFs—BDNF, NGF, NT-3, NT-4/5, CNTF and GDNF—are detected in adult RGCs, which have also been shown to express the respective NTF receptors [3,77,78,79,80,81,82,83]. Moreover, both target neurons and astrocytes have been shown to synthesize and secrete a variety of NTFs, such as BDNF in the SC and dLGN [72,84,85,86,87,88,89]. A logical corollary was the implementation of tracing experiments. Indeed, application of radiolabeled BDNF to the SC revealed retrograde transport of the labeled NTF towards the RGC soma, whereas this transport was substantially diminished in rodent and dog glaucoma models [90,91]. It should be mentioned that these radioautography experiments demonstrate transport failure by observing accumulation of the radiolabeled BDNF at the optic nerve head, as compared to anterior (retina) and posterior (axon) sites. Therefore, these studies did not really corroborate axonal transport disruption, but rather NTF trapping at the optic nerve head. Yan et al. [92] injected radiolabeled GDNF into a target brain area and observed significantly accumulated labeling in the retina contralateral to the target injection side. Moreover, by co-injecting a surplus of unlabeled GDNF, a threefold decrease in detected retinal radiolabeling was observed. This finding hints that the transport of GDNF is receptor mediated [92]. In another interesting study, Takihara and colleagues [93] administered fluorescently tagged BDNF to cultured rat RGCs and were able to follow axonal transport via live-cell imaging. The labeled BDNF was shown to move along the axon in an anterograde and retrograde direction, hence unveiling that RGCs are able to transport at least one NTF. When applying colchicine, a drug causing microtubule disruption, movement of the labeled BDNF was significantly inhibited. This finding documents the involvement of microtubules in axonal transport of BDNF. An important notice of this study is that RGCs were harvested from three-day old rat pups and live-cell imaging was performed at P16, which is still a developmental stage in rats [93]. In short, retrograde transport of at least two exogenously supplied NTFs—BDNF and GDNF—was shown in naive settings and found to be obstructed in animal glaucoma models. The transport is most likely receptor-mediated and involves microtubule transport. Of note, although these experiments illustrate that NTFs can make it to the retina, they fail to prove that endogenous NTFs really do travel from the brain towards the retina. As such, cold hard evidence ascertaining that retrograde transport is a prominent route for NTFs in the retina is still missing.

### 4.3. NTF Deprivation in the Glaucomatous Retina

If NTF deprivation is indeed prompting RGCs into apoptosis, one can expect a decreased level of NTFs in the eye after glaucomatous injury. Indeed, decreased BDNF levels were observed in aqueous humor, blood serum and tears of human patients [94,95,96]. Nevertheless, studies on NTF levels in the eyes of glaucomatous animals are contradictory; some indeed show a reduced presence, others report no alteration or even an enhanced expression. A brief overview is given in a review by Pietrucha-Dutczak et al. [97]. Importantly, comparing these studies in a straightforward way is rather difficult as they make use of different glaucoma models, species, quantification techniques, timing and regions of interest. This region of interest—e.g., the target area, the optic nerve head, the RGCs (layer) or the whole retina—is of great importance since unique RGC responses could be diluted by injury responses from other retinal cells. Indeed, in addition to target-derived supply, NTFs can be provided via local production in an autocrine—i.e., originating from RGCs—or paracrine way —i.e., originating from other retinal cells than RGCs, like Müller and amacrine cells [8,98,99,100,101,102,103,104]. Hence, the upregulation of local support can temporarily mask and possibly reverse a target-derived NTF depletion.

## 5. NTFs as Neuroprotective Therapy in Glaucoma: What Information Are We Missing?

The NTF deprivation theory in glaucoma has emerged from the general concept that neurons depend on their target structure for proper functioning and survival. It accredits an important role to diminished levels of NTFs in the retina when RGCs are dying after axonal transport deficits. From this perspective, the NTF deprivation theory pinpoints a specific shortage of retrogradely transported target-derived NTFs and not a general lack of trophic support. However, NTF deprivation might also occur locally, since retinal glial cells are known to undergo morphological and functional changes in response to injury, which might alter their NTF production and secretion, contributing to a local deprivation of trophic support [105,106,107,108]. Note that this clearly pinpoints the need for in vivo studies regarding the NTF mechanism of action, since in vitro studies often rule out the contribution of other (retinal) cells. Additionally, decreased levels of BDNF are also observed in patients with age-related macular degeneration, an eye disease that is not characterized by axonal transport deficits [109]. This again reinforces the statement that alterations in local NTF supply and/or function may be an additional contributing factor in the progression of glaucoma and should not be overlooked. Of note, researchers have not yet been able to distinguish local and target-derived NTFs in the retina. As such, one of the major unsolved questions in the NTF deprivation theory is: is the observed decrease in retinal NTF levels due to a diminished supply of target-derived or local NTFs, or both?

**Figure 2:**
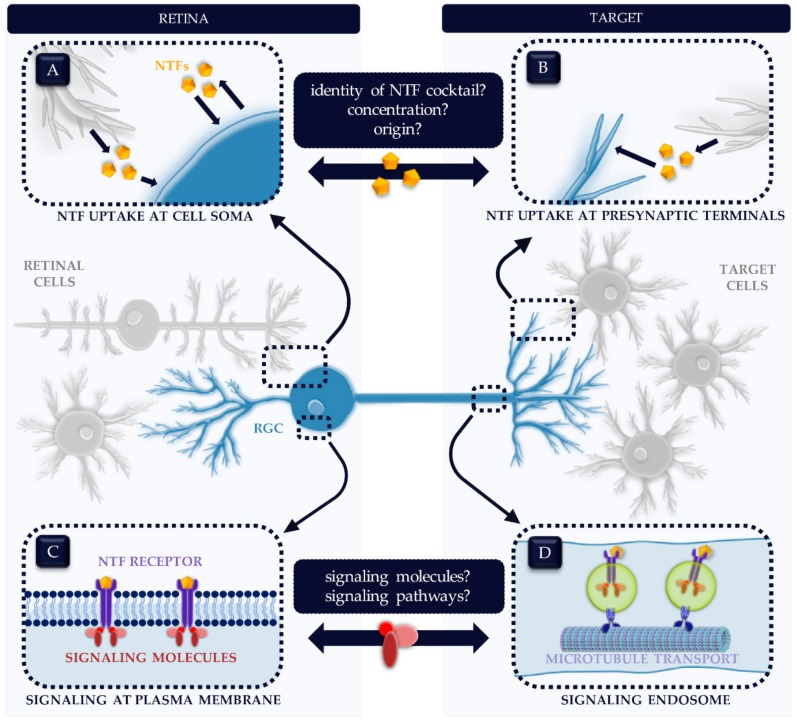
Gaps in the fundamental knowledge of neurotrophic factors (NTFs) in the adult visual system. (**A**,**B**) Many fundamental questions regarding the NTF cocktail essential for retinal ganglion cell (RGC) survival remain unanswered. Precisely which NTFs are involved? Is the interplay between two or more NTFs a bare necessity? If so, what is the ideal ratio of each NTF in the cocktail? Do vital NTFs originate from local sources or from target areas, or both; and from which specific cell type—i.e., neurons and/or glial cells? (**C**,**D**) Also on cellular level, a number of research questions are yet to be unraveled regarding the NTF mechanism of action. How do NTFs function upon receptor binding? What is the complete array of involved signaling molecules and pathways? (**A**,**C** versus **B**,**D**) All of the abovementioned parameters might differ depending on the site of NTF presentation: at the cell soma (**C**) or at the presynaptic terminals (**B**,**D**). For example, recruitment of downstream signaling molecules varies upon the location where the signaling machinery is switched on: the cell soma for retinal NTFs (**C**) versus the distal axon for target-derived NTFs (**D**).

An evident corollary of the observed NTF deprivation and the accompanying theory was exploring the neuroprotective potential of local delivery of NTFs. In the past decades, a myriad of studies was dedicated to scrutinizing this potential therapy. NTFs were supplemented via numerous ways such as intraocular injection, topical administration, gene therapy, stem cell transplantation, transgenic recombinase strategies, etc. These studies repeatedly demonstrated an enhanced RGC survival in different animal models of glaucoma. Hence, these experiments support the idea that RGCs indeed suffer from NTF deprivation and that locally supplementing NTFs aids them in their struggle for survival. Direct supplementation of NTFs to glaucomatous RGCs undeniably has an enormous neuroprotective potential yet this therapy is still in its infancy. This might be due to the hiatuses in our fundamental knowledge of NTFs in the adult visual system [110], as depicted in Figure 2. In the following Sections, we will discuss the major shortcomings of present-day NTF therapy efforts and reflect on associated knowledge gaps in the basic understanding of NTFs.

### 5.1. First Shortcoming of NTF Therapy Efforts: The Transient Effect

A common finding in all NTF supplementation studies is the temporary effect. Local NTF supplementation seems to delay rather than prevent the onset of RGC degeneration, and thus falls short in promoting sustained survival. Efforts to prolong the effect—by repeated injections or by sustained delivery mechanisms—have failed, possibly due to receptor downregulation (desensitization) [86,111,112,113,114,115,116]. Indeed, the expression of NTF receptors is altered by glaucomatous injury, thus complicating exogenous supplementation of NTFs as glaucoma therapy. To circumvent this problem, present studies are investigating the possibility to implement both BDNF and its corresponding receptor in gene therapy constructs and initial results look promising [117,118]. Another way to bypass fluctuating receptor availability is to directly influence a downstream actuator in the signaling pathway of the NTF [119,120]. A handful of studies report striking RGC neuroprotection in rat models of glaucoma with viral vector delivery of a pro-survival pathway activator—e.g., MEK1, Bcl-X_L_ and BAG1 [121,122,123]—or a pro-apoptosis pathway inhibitor—e.g., BIRC4, p35 and CPP32-like caspase inhibitor [124,125,126]. However, these reports again document a temporary effect, which might arise from targeting only one specific signaling pathway. Neuroprotection does not only entail avoiding the process of apoptosis, it should also secure the recovery of the neuron towards homeostasis [127]. The pleiotropic effects and the resulting broad spectrum of functions might exactly be the power of NTFs, although at the same time also the limitation in clinical applications due to undesirable side effects [119,120]. Hence, researchers are exploring (ant)agonists that selectively mimic a specific outcome of NTFs, without any side effects of prolonged or excessive NTF delivery [79,119]. For example, neurotrophins can bind two distinct types of receptors: the tropomyosin related kinase (Trk) and the p75 neurotrophin (p75^NTR^) receptors. Although highly simplified, one can say that Trk receptor binding is neuroprotective, whereas p75^NTR^ receptor binding can be both neuroprotective and neurodestructive [128]. As such, several studies have focused on selective Trk receptor agonists, which mimic the function of NTFs without binding to the p75^NTR^ receptor. Examples of such agonists are 7,8-DHF, 1D7 and NGF-C [129,130,131]. 7,8-DHF showed neuroprotective effects in a primary rat RGC culture after excitotoxic and oxidative stress [131], whereas 1D7 and NGF-C conveyed significant in vivo neuroprotection after ONI and episcleral vein cauterization in adult rats [129,130]. These reports again emphasize the importance of understanding the molecular mechanisms underlying NTF function, as neuroprotection might rely on both the activation and inhibition of specific pathways.

### 5.2. Second Shortcoming of NTF Therapy Efforts: The Focus on Monotherapy

A second major shortcoming of most experimental NTF therapies is the focus on a single NT, whilst combinatorial approaches might exceed the neuroprotective potential of NTF monotherapy. Moreover, RGCs represent a diverse population; a single-cell transcriptomics experiment has revealed more than 40 RGC subtypes [132]. As such, it might not be surprising that NTF requirements differ for distinct RGC subpopulations and multiple NTFs should act in concert to protect the complete array of RGCs. Already in 1999, it was shown that intravitreal injection of a BDNF and GDNF mixture led to enhanced RGC survival compared to separate BDNF or GDNF injections in an ONI model [92]. This experiment was later confirmed by Koeberle et al. [81], who showed similar results with co-administration of BDNF and neurturin, another NTF belonging to the TGF-β superfamily. Furthermore, using the same injury model, intravitreal injection of fibroblasts overexpressing single or combined BDNF, NT-3 and basic fibroblast growth factor (bFGF, member of the non-neuronal factor superfamily) was compared. Triple NTF expressing fibroblasts significantly boosted RGC survival in comparison to single NTF expressing fibroblasts [133]. Likewise, dual injection of neural stem cells releasing GDNF or CNTF showed a synergistic effect after ONI [134]. A very recent study of Kitamura et al. [135] compared the in vivo effects of single and combined NTF administration on RGC survival in adult rats after ONI. Once more, they showed that the combinatorial approach was the most effective one. Of note, besides NT-4, they studied rather unconventional NTFs: tauroursodeoxycholic acid and citicoline [135]. In sharp contrast, using a laser-induced glaucoma model, Pease et al. [136] were unable to show an additive effect of dual injection of viral vectors overexpressing BDNF or CNTF on RGC survival. This might be due to an unsuitable concentration of one or both NTFs. Indeed, the therapeutic effect of NTFs—either alone or acting in concert—is dose-dependent [92,135,136,137]. Another reason might be a high degree of overlapping pathways induced by both NTFs. As aforementioned, signaling pathways underlying NTF action are not fully unraveled, which is clearly hampering the addition of NTF to the neuroprotective therapeutic armamentarium. This might also explain why 20 years after the first promising results of a combinatorial approach, there is still no major progress in this field. Both the identity, concentration as well as the downstream effectors of NTFs that are essential for long-term neuroprotection are unknown.

### 5.3. Third Shortcoming of NTF Therapy Efforts: Superfluous Supplementation

The unknown optimal concentration gives rise to a third shortcoming that might explain the transient neuroprotective effect: an unnatural surplus of NTF levels. Besides the injury itself, receptor desensitization might also be caused by a NTF oversupply and leaves RGCs unresponsive to repetitive NTF therapy. On the other hand, excessive levels of NTFs might induce different pathways as compared to those triggered by endogenous NTF levels. To sum up, NTF oversupply might provoke undesired effects and/or receptor downregulation, which might be as detrimental as a shortage of NTFs. Circumventing a surplus of NTFs could be achieved by artificially enhancing neuronal activity in the retina. This hypothesis is based on the tight linkage between neuronal activation and release of NTFs [138,139,140]. The stimulation of neuronal activity might induce a more endogenous-like, and thus more physiologically relevant stimulation of NTF expression as compared to artificial supplementation. Ocular electrical stimulation (ES) has shown promising results for neuroprotective therapy in retinal and optic nerve diseases as reviewed by Sehic et al. [141] and Manthey et al. [142]. Here, we provide a brief overview of studies investigating ES and NTF involvement in the visual system. After ES, cultured Müller cells from postnatal rats are shown to upregulate BDNF [143,144], CNTF [144] and insulin-like growth factor 1 (IGF-1) [145]. Upregulation of mRNA and protein expression levels of the anti-apoptotic molecule Bcl-2 and the NTFs CNTF and BDNF, and a downregulation of the pro-apoptotic molecule Bax was also observed in vivo after transcorneal—i.e., via a contact lens with electrodes—ES in adult rats. Remarkably, Bcl-2 and CNTF expression was specifically enhanced in the Müller cells [146]. Further, bFGF was also shown to be upregulated after subretinal ES in a dystrophic rat retinal degeneration model [147]. Last, Morimoto et al. [148] demonstrated a neuroprotective effect of transcorneal ES in adult rats. After subjecting those rats to ONI, ES was able to lever RGC survival up to 85% one week after ONI, as compared to 53% in the control retinas. Interestingly, they hypothesized that this enhanced RGC survival might be caused by an upregulation of NTFs and performed reverse transcription polymerase chain reaction (RT-PCR) on four different NTFs; BDNF, CTNF, bFGF and IGF-1. Surprisingly, the only altered NTF of those interrogated was IGF-1, of which mRNA and protein levels were increased. Combined application of ES with systemic administration a IGF-1 receptor blocker then nearly completely abolished the previously observed neuroprotective effect [148]. Notably, techniques most often used in these studies are RT-PCR and Western blot, both requiring prior knowledge about the identity of the mRNA/proteins to be investigated, and thus ignoring other possible molecules involved. Willmann et al. [149], however, performed a whole genome-wide expression profiling and looked at up- and downregulated pathways four hours after transcorneal ES in naive adult rats. Two of the most prominent findings in their dataset are the downregulation of the anti-apoptotic factor Bax and the fact that there was no differential expression of the previously mentioned NTFs [149]. Hence, their data combined with the aforementioned differences in NTF expression after ocular ES hint that the set of mRNA/protein changes might depend on the time point of RNA isolation and the injury model and might include differential expression of NTFs other than the usual suspects.

Notwithstanding the clear knowledge gap, and following the neuroprotective effect of transcorneal ES in different optic neuropathy models in rats [148,150,151], transcorneal ES has already been tested in human patients with traumatic and ischemic optic neuropathy. For the majority of the patients, transcorneal ES ameliorated visual acuity without any major complications [152]. Similarly, several clinical trials with repetitive transorbital alternating current stimulation in patients with optic nerve damage/lesions have shown partial restoration of vision [153,154,155,156]. Some studies reported enhanced visual acuity and field size in half of the patients [155], others showed only improved visual field, not visual acuity [156]. At present, a clinical pilot study is ongoing in which the efficacy and feasibility of repetitive transorbital alternating current stimulation in glaucoma patients is being examined (NCT03188042, source: www.clinicaltrials.gov, last access date: 30 August 2019).

### 5.4. Fourth Shortcoming of NTF Therapy Efforts: A Possible Difference between Local and Target-Derived NTFs

Another theory to explain the temporary effect of NTF supplementation states that local NTF support is both necessary and sufficient to shield the RGCs from initial damage, but target-derived support must come into play for long-term neuroprotection. This hypothesis is supported by the study of Weber et al. [157], who showed that applying BDNF both to the eye and to the brain in adult cats subjected to ONI provoked prolonged and enhanced RGC survival compared to a sole eye treatment. Astonishingly, the resulting survival levels observed with this dual approach were—even after two weeks—comparable to naive RGC counts [157]. Aforementioned target lesion/removal experiments also seem to espouse this hypothesis, as significant RGC loss was only detected after several weeks or months post-lesion. However, it is important to mention that although target ablation did not cause immediate RGC death, it was shown to abruptly impair RGC functionality [53]. In light of these observations, local NTF support may overcome chronical loss of target support by safeguarding RGCs from apoptosis at early stages. However, RGCs are still in need of target-derived NTFs to survive and preserve their full functionality. If this hypothesis holds true, NTFs from different sources could present distinct physiological roles.

A possible difference between local and distal NTFs are the diverse signaling outcomes that can be shaped by differences in receptor localization: receptors anchored to the plasma membrane versus internalized receptor complexes traveling alongside the axon [158,159,160]. The generally accepted mechanism for NTF-receptor trafficking towards the cell body is the signaling endosome model [38]. This model was first identified for NGF signaling [161,162] and was later confirmed for BDNF [163]. At the presynaptic terminals of the neurons, NTFs bind to their membrane receptors. After endocytosis, a vesicle containing the NTF-receptor complex is formed, which starts recruiting downstream signaling molecules, thereby giving rise to the so-called signaling endosome, which is retrogradely transported to the cell soma via microtubule-mediated dynein-dynactin active transport (Figure 2). Ligand-receptor complexes can be sustained in the signaling endosome, hence leading to receptor activation, attraction of downstream molecules and stimulation of transcriptional responses prior to arrival of the endosome at the cell body [29,38,75,160,164,165,166,167]. This long-distance activation of transcriptional events is one of the most striking aspects of NTF signaling [75]. On the contrary, locally secreted NTFs directly bind their receptor at the RGC plasma membrane, hence provoking the initiation of signaling pathways in the RGC soma [8]. Distinct sites of receptor activation—either at the plasma membrane or within axonal vesicles—are known to induce specialized signaling pathways [29,158,159,168]. Although not extensively, this has also been shown in the visual system. In the embryonic retinotectal visual system of *Xenopus laevis*, Lom et al. [169] showed a complementary effect of local and target-derived BDNF delivery on RGC dendritic arborization. Although in another model system (spinal nerve) and in vitro, NTF stimulation at the cell soma was shown to activate two downstream effectors ERK1/2 and ERK5 of the MAPK pathway, whereas NTF presentation at the distal axon only controlled ERK5 [170]. Building on this observation, Van Oterendorp et al. [171] were able to validate this finding in vivo in adult rats. Applying BDNF to the RGC soma upregulated ERK1/2 and ERK5, compared to a sole ERK5 upregulation upon BDNF stimulation at the RGC axon ending [171]. Discrepancies in local versus target-derived NTF signaling are just emerging and further elucidation might be the next step in successful NTF therapy for glaucomatous neuroprotection.

### 5.5. Tackling the Shortcomings of NTF Therapy Efforts

**Figure 3:**
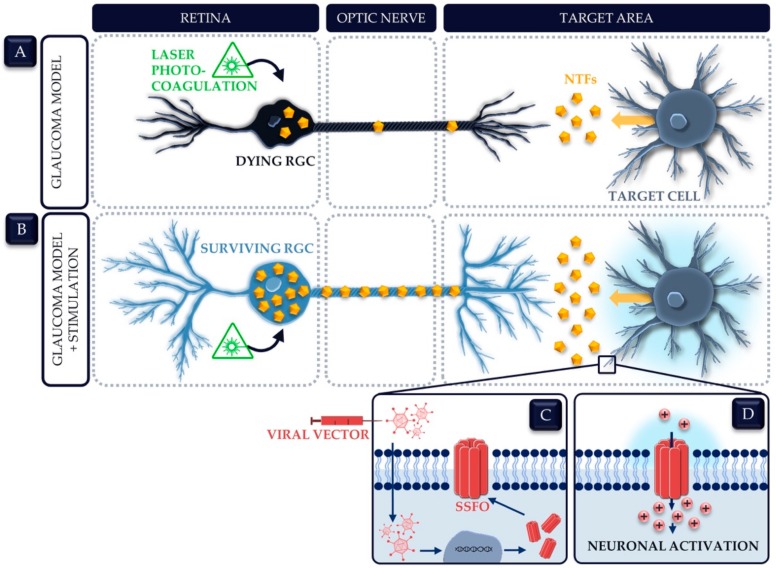
Does optogenetic stimulation of a target brain area trigger an upregulation of target-derived neurotrophic factors (NTFs) vital for retinal ganglion cell (RGC) survival after glaucomatous injury? (**A**) In glaucomatous conditions—e.g., after laser photocoagulation of the rodent eye—RGCs are struggling to survive, supposedly and at least partly due to NTF deprivation. (**B**) Applying the same injury model whilst chronically stimulating neuronal activity in the principal RGC target area in the brain, enables the RGCs to survive these stressing conditions. This observed neuroprotective effect might be attributable to an increased production and transport of target-derived NTFs as a result of target stimulation. (**C**,**D**) One possible stimulation modality is optogenetics, in which genes encoding for light sensitive modulators are induced via viral vector technology (**C**). The prototype modulator for the stimulation of neuronal activation is a microbial cation channel—e.g., a stabilized step function opsin (SSFO). Light irradiation will open the channels and cause a resulting increase in cytosolic cations, which drives neuronal depolarization and subsequent neuronal spiking (**D**).

In a recent publication, we tried to overcome the four abovementioned shortcomings in order to increase RGC survival in a glaucomatous retina. Instead of a continuous and possible overkilled supplementation of a single NTF, we opted to focus on controlled stimulation of neuronal activity. As such, we explored whether controlled stimulation of neuronal activity in the main retinofugal murine target area, i.e., the SC, conferred neuroprotection in a glaucoma model [172]. To attain such a protracted stimulation, we used optogenetics due to its superior spatial resolution in comparison with other stimulation modalities. In short, a light sensitive microbial cation channel—a stabilized step function opsin (SSFO)—was introduced in the SC via viral vector technology. Blue light irradiation initiated the opening of the channel and subsequent influx of positive charges into the cell, causing a depolarization and neuronal firing. Next, glaucomatous RGC death was induced via laser photocoagulation of the perilimbal and episcleral veins. Repeated optogenetic stimulation was performed twice a day, starting the day before laser treatment until 14 days post injury. Following RGC quantification, the optogenetically stimulated group showed a significant increase in surviving RGCs (90%) as compared to the unstimulated group (74%). Thereby, we showed that repeated stimulation of neuronal activity in the SC is neuroprotective for RGCs subjected to glaucomatous injury, plausibly by an increase in the production and transport of a vital target-derived NTF cocktail (Figure 3) [172]. Notably, although we focused on neuronal activation, we believe that optogenetic stimulation might affect the complete astroglial-neural network. Astrocytes are known to sense the activity of their surrounding neurons and undergo functional changes in response to increased activity [173]. This is of paramount importance since astrocytes present an additional source of NTFs and as such, could amplify the neuroprotective effect of neuronal stimulation [72,89].

Ongoing work includes the examination of the mechanism by which optogenetic target stimulation mediates retinal neuroprotection. By tagging newly synthesized proteins in the SC, retrogradely transported factors will be identified via proteomic approaches. Similarly, we are investigating which signaling pathways in the retina are specifically up-/downregulated in our setup via a single-cell transcriptomics experiment. This way, signaling actuators will be uncovered in an unbiased way, in contrast to standard techniques—e.g., Western blot, RT-PCR, enzyme-linked immunosorbent and activity assays—all of which require prior knowledge. Moreover, comparative transcriptomics after target and local stimulation—either by NTF supplementation or by ocular ES—will allow direct comparison of locally versus target-mediated neuroprotective signaling pathways. Ultimately, appropriate therapeutic manipulation of survival factors and/or downstream signaling pathways important for target-derived RGC survival might result in the design of an efficient, long-term neuroprotective treatment. In our study, we exclusively stimulated neuronal activity in the target area whilst excluding RGC activation to bring in a verdict around the importance of target areas in RGC neuroprotection [172]. However, as it has been shown that local stimulation also entails neuroprotective effects—see Section 5.3—, both target and local stimulation might be employed in synergy to lever RGC survival.

## 6. Fishing for Interspecies Differences in the NTF Deprivation Theory

Frequently used injury models to study the NTF deprivation theory are ONI models—in which the optic nerve is either crushed or transected [174]. Strikingly, the onset, progression, as well as the extent of RGC death, lucidly differ in various animal species after ONI [175,176]. A remarkable observation is the apparent linkage between RGC neuroprotection and axonal regeneration. Species capable of regrowing their RGCs axons and restoring synaptic connections with their target neurons in the brain—amniotes and reptiles—seem to show considerably less RGC death compared to species who fail to repair/regrow the damaged axon—birds and mammals [177,178,179,180,181,182,183,184,185,186,187,188]. For example, comparing RGC survival in rodents versus teleost fish after ONI reveals a striking difference: studies report extensive RGC loss (~75–90%) in rodents [56,86,176,188,189,190,191,192,193,194,195,196,197,198,199], whereas close to all piscine RGCs seem to withstand the injury [200,201,202,203,204]. In a recent publication, we have shown that adult zebrafish show a remarkably swift and robust regrowth of RGC axons to their principal target area—i.e., the optic tectum—after optic nerve crush. Axons reach the optic tectum 5 days post-lesion and complete reinnervation is accomplished at 10 days post crush [205], which is in agreement with other reports [203,206,207]. On the contrary, axonal regeneration is slower, less robust and sometimes only partially achieved—e.g., due to axonal misrouting—in adult amphibians. Remarkably, this seems to correlate with higher RGC loss (40–80%) after ONI [177,178,181,182,184,204,208,209,210,211,212]. These observations collectively hint towards the hypothesis that target reinnervation is a bare necessity for RGC survival and that its timing and robustness determines the extent of RGC neuroprotection. This postulation perfectly reconciles with the NTF deprivation theory: RGCs capable of reconnecting to their target structures regain their vital target-derived NTF support, which strengthens their resistance to the stressing conditions caused by ONI. In sharp contrast, adult mammalian and avian RGCs lacking regenerative capacities irretrievably lose their target (NTF) support and as such surrender to the injury.

Besides differences in the re-establishment of target-derived NTF support, there could also be interspecies differences in local NTF signaling after injury. NTFs in RGC axonal regeneration has been extensively studied in fish [213], but their role in teleost RGC neuroprotection has been overlooked. This is rather surprising since neuroprotection is a prerequisite for successful regeneration. Notwithstanding, there are some indications that at least one NTF might play a role in the observed interspecies difference in neuroprotection. The research group of dr. Kato investigated and compared the expression of anti- and pro-apoptotic molecules of the PI3K/Akt signaling pathway in the early stages after ONI in the goldfish and rat retina [201,214,215]. In the goldfish retina, there was an increase in the anti-apoptotic molecules p-Akt, p-Bad and Bcl-2, whereas the pro-apoptotic molecule Bax was unaltered and caspase 3 was decreased [214]. In sharp contrast, an opposite reaction was observed in the rat retina: a decrease in the anti-apoptotic molecules and an increase of the pro-apoptotic molecules [215]. Interestingly, they also observed a contrast in the expression of IGF-1, which decreased in rodent RGCs yet increased in piscine RGCs [201]. A corollary of this interspecies difference was to examine whether intraocular injection of IGF-1 was able to rescue RGCs in rats after ONI. Indeed, as mentioned above, a significant neuroprotective effect of IGF-1 has been found in several studies. What is very interesting, however, is that a strong upregulation of the IGF-1 protein was observed in the endfeet of the Müller glia, located in the ganglion cell layer [148]. This suggests that differences in Müller glia actions might feed differences in neuroprotective capacity of mammals versus teleost fish [216], an assumption that is plausible as piscine Müller glia are shown to be reprogrammed into multipotent retinal progenitor cells with true stem cell characteristics after retinal injuries. All these data then hint towards a neuroprotective environment surrounding the RGCs, instead of endogenous neuroprotective capacities of RGCs themselves. Hence, the secretion of survival factors arising from neighboring cells might help RGCs to sustain injury. This notion can be illustrated by the striking differences in nitric oxide production, which can either be neuroprotective or cytotoxic depending on the resulting intracellular nitric oxide concentration, in teleosts versus mammals [217,218]. After ONI, a comparable upregulation of nitric oxide synthase proteins was observed in goldfish [218] and rat [219,220] RGCs. Conversely, a remarkable difference was spotted in the Müller glia. Whilst rat Müller glia were shown to have elevated levels of nitric oxide synthase after ONI [220], the activity of nitric oxide synthase was unaltered in goldfish Müller glia [218]. At first sight, nitric oxide might look far-fetched in this NTF story. However, multiple studies have demonstrated an interplay between nitric oxide synthase and NTFs. Studies on postnatal rat hippocampal neurons showed, for instance, that artificially raising nitric oxide in the close environment of neurons by the application of a nitric oxide donor causes inhibition of BDNF [140,221] and NT-3 secretion [140].

Besides IGF-1 and nitric oxide synthase, other cell survival/death signals have been shown to be differentially expressed between rodents and fish after ONI, at early stages. Examples are heat shock protein 70 (Hsp70) [201,222], neuroglobin [223,224] and semaphorin-3A [225,226]. Of course, there are more differentially expressed molecules in fish and rodents after ONI than cited here, as described by Ogai et al. [227]. However, we choose to only quote findings occurring very rapidly after injury, since the molecular events underlying neuroprotection and neuroregeneration can no longer be separated after a few days. Moreover, it might still be that the observed RGC survival in teleost fish is unrelated to NTFs—e.g., due to the presence of other protection mechanisms that prevail in teleost retinas after ONI. Either way, the opposing patterns observed in NTFs and other possible survival actors in rodent and fish RGCs post ONI present an excellent opportunity to investigate novel neuroprotective routes. What are the factors that determine the fate of the RGC? What makes that fish RGCs withstand a damaging environment, whereas rodent RGCs just seem to surrender? We believe that comparative research, specifically focusing on the differences in innate neuroprotection, might create new avenues for future NTF research.

## 7. Concluding Remarks and Future Perspectives

The theory of NTF deprivation is a very attractive and generally accepted hypothesis to explain why RGCs undergo apoptosis after glaucomatous injury. Even though many (in)direct observations are in favor of the theory, we are far from a complete understanding of the exact NTF functioning. This is not surprising as NTFs and their signaling in the healthy and diseased visual system—and generally in the nervous system—present a highly complex story. A myriad of NTFs have been appointed as lead neuroprotective molecules, mainly by testing their potency via exogenous supplementation in animal glaucoma models. Most studies center their research around a small group of NTFs, although chances are high that certain NTFs and maybe even molecules not yet appointed to the NTF family are as powerful or more promising neuroprotectants. The majority of past research outcomes was based on techniques such as RT-PCR and Western blotting, which rely on prior knowledge. Hence, identification of novel factors and/or complete signaling pathways is ruled out. Besides, most research focusses on single NTF administration, whilst a combinatorial approach could be more beneficial. In summary, both the ingredients as well as the correct proportions of the vital survival cocktail in the adult visual system remain elusive. Determining the optimal concentration of the NTFs is of paramount importance since an insufficient quantity will not rescue the RGCs, whereas an oversupply might cause receptor desensitization and undesirable side effects. The combination of all of the aforementioned hindrances might explain why the efficacy of NTFs as neuroprotective strategy in glaucoma has been disappointing. To augment the potential of NTF therapy and to advance present-day NTF research, we suggest exploring the complete spectrum of underlying signaling pathways in greater detail. Given the recent advances in the omics field, fundamental research tackling this lack of knowledge could lever and truly embark NTF based therapies. Without doubt, single-cell transcriptomics might come to the rescue. This technique does enable the unraveling of the exact machinery and downstream effects of NTFs on RGCs in complex in vivo situations and may provide a novel means to improve our understanding of the role of other retinal cells—i.e., Müller glia—in NTF signaling in the retina. Moreover, comparative studies between rodent and piscine RGC neuroprotection after ONI might reveal additional avenues towards neuroprotective strategies. Besides, we enhearten to focus novel studies on the relationship between the eye and the brain and, in particular, to focus on target-derived NTFs. Whether a differential signaling from local and target-derived NTFs is essential in (glaucomatous) optic neuropathies still needs to be shown but is highly important for the future design of effective NTF therapies. In order to add NTF approaches to the treatment repertoire of neurodegenerative disease in general and glaucoma in particular, it is of the utmost importance to enhance the comprehension of these tiny, but vital molecules.

## Abbreviations

BDNF	Brain-derived neurotrophic factor
bFGF	Basic fibroblast growth factor
CNTF	Ciliary nerve trophic factor
dLGN	Dorsal lateral geniculate nucleus
ES	Electrical stimulation
GDNF	Glial cell-derived neurotrophic factor
Hsp70	Heat shock protein 70
IGF-1	Insulin-like growth factor
JAK/STAT	Janus kinase/signal transducers and activators of transcription
JNK	c-Jun N-terminal kinase
MAPK/ERK	Mitogen-activated protein kinase/extracellular-signal-regulated kinase
NF-κB	Nuclear factor kappa-light-chain-enhancer of activated B
NGF	Nerve growth factor
NT-3	Neurotrophin-3
NT-4/5	Neurotrophin-4/5
NT-6	Neurotrophin-6
NT-7	Neurotrophin-7
NTF	Neurotrophic factor
ONI	Optic nerve injury
p75^NTR^	p75 neurotrophin
PI3K/AKT	Phosphoinositol-3 kinase/protein kinase B
PLC-γ	Phospholipase C- γ
RGC	Retinal ganglion cell
RT-PCR	Reverse transcription polymerase chain reaction
SC	*Superior colliculus*
SSFO	Stabilized step function opsin
TGF-β	Transforming growth factor β
Trk	Tropomyosin related kinase

## References

[1] Foster P.J. (2002). The definition and classification of glaucoma in prevalence surveys. Br. J. Ophthalmol..

[2] Chua B., Goldberg I. (2010). Neuroprotective agents in glaucoma therapy: Recent developments and future directions. Expert Rev. Ophthalmol..

[3] Almasieh M., Wilson A.M., Morquette B., Cueva Vargas J.L., Di Polo A. (2012). The molecular basis of retinal ganglion cell death in glaucoma. Prog. Retin. Eye Res..

[4] Foldvari M., Chen D.W. (2016). The intricacies of neurotrophic factor therapy for retinal ganglion cell rescue in glaucoma: A case for gene therapy. Neural Regen. Res..

[5] Nickells R.W. (2007). From ocular hypertension to ganglion cell death: A theoretical sequence of events leading to glaucoma. Can. J. Ophthalmol..

[6] Nickells R.W. (2012). The Cell and Molecular Biology of Glaucoma: Mechanisms of Retinal Ganglion Cell Death. Investig. Opthalmol. Vis. Sci..

[7] Vrabec J.P., Levin L.A. (2007). The neurobiology of cell death in glaucoma. Eye.

[8] Johnson E.C., Guo Y., Cepurna W.O., Morrison J.C. (2009). Neurotrophin roles in retinal ganglion cell survival: Lessons from rat glaucoma models. Exp. Eye Res..

[9] Knox D.L. (2007). Optic Nerve Hydropic Axonal Degeneration and Blocked Retrograde Axoplasmic Transport. Arch. Ophthalmol..

[10] Burgoyne C.F. (2011). A biomechanical paradigm for axonal insult within the optic nerve head in aging and glaucoma. Exp. Eye Res..

[11] Quigley H.A., Davis E.B., Anderson D.R. (1977). Descending optic nerve degeneration in primates. Invest. Ophthalmol. Vis. Sci..

[12] Ceni C., Unsain N., Zeinieh M.P., Barker P.A., Lewin G.R., Carter B.D. (2014). Neurotrophins in the Regulation of Cellular Survival and Death. Neurotrophic Factors.

[13] Fahy E.T., Chrysostomou V., Crowston J.G. (2016). Mini-Review: Impaired Axonal Transport and Glaucoma. Curr. Eye Res..

[14] Millecamps S., Julien J.-P. (2013). Axonal transport deficits and neurodegenerative diseases. Nat. Rev. Neurosci..

[15] Bartus R.T., Johnson E.M. (2017). Clinical tests of neurotrophic factors for human neurodegenerative diseases, part 1: Where have we been and what have we learned?. Neurobiol. Dis..

[16] Zhang K., Hopkins J.J., Heier J.S., Birch D.G., Halperin L.S., Albini T.A., Brown D.M., Jaffe G.J., Taoj W., Williams G.A. (2011). Ciliary neurotrophic factor delivered by encapsulated cell intraocular implants for treatment of geographic atrophy in age-related macular degeneration. Proc. Natl. Acad. Sci. USA.

[17] Lanni C., Stanga S., Racchi M., Govoni S. (2010). The Expanding Universe of Neurotrophic Factors: Therapeutic Potential in Aging and Age-Associated Disorders. Curr. Pharm. Des..

[18] Levy Y.S., Gilgun-Sherki Y., Melamed E., Offen D. (2005). Therapeutic Potential of Neurotrophic Factors in Neurodegenerative Diseases. BioDrugs.

[19] Caminos E., Becker E., Martín-Zanca D., Vecino E. (1999). Neurotrophins and their receptors in the tench retina during optic nerve regeneration. J. Comp. Neurol..

[20] McMahon S., Murinson B. (2005). Therapeutic Potential of Neurotrophic Factors. BioDrugs.

[21] Nilsson A.S., Fainzilber M., Falck P., Ibáñez C.F. (1998). Neurotrophin-7: A novel member of the neurotrophin family from the zebrafish. FEBS Lett..

[22] Park B.-C., Tibudan M., Samaraweera M., Shen X., Yue B.Y.J.T. (2007). Interaction between two glaucoma genes, optineurin and myocilin. Genes to Cells.

[23] Sippl C., Bosserhoff A.K., Fischer D., Tamm E.R. (2011). Depletion of optineurin in RGC-5 cells derived from retinal neurons causes apoptosis and reduces the secretion of neurotrophins. Exp. Eye Res..

[24] Nowak A., Majsterek I., Przybyłowska-Sygut K., Pytel D., Szymanek K., Szaflik J., Szaflik J.P. (2015). Analysis of the Expression and Polymorphism of APOE, HSP, BDNF, and GRIN2B Genes Associated with the Neurodegeneration Process in the Pathogenesis of Primary Open Angle Glaucoma. Biomed. Res. Int..

[25] Pasutto F., Matsumoto T., Mardin C.Y., Sticht H., Brandstätter J.H., Michels-Rautenstrauss K., Weisschuh N., Gramer E., Ramdas W.D., van Koolwijk L.M.E. (2009). Heterozygous NTF4 Mutations Impairing Neurotrophin-4 Signaling in Patients with Primary Open-Angle Glaucoma. Am. J. Hum. Genet..

[26] Nowak A., Szaflik J.P., Gacek M., Przybylowska-Sygut K., Kamińska A., Szaflik J., Majsterek I. (2014). BDNF and HSP gene polymorphisms and their influence on the progression of primary open-angle glaucoma in a Polish population. Arch. Med. Sci..

[27] Liu Y., Liu W., Crooks K., Schmidt S., Allingham R.R., Hauser M.A. (2010). No Evidence of Association of Heterozygous NTF4 Mutations in Patients with Primary Open-Angle Glaucoma. Am. J. Hum. Genet..

[28] Rao K.N., Kaur I., Parikh R.S., Mandal A.K., Chandrasekhar G., Thomas R., Chakrabarti S. (2010). Variations in NTF4, VAV2, and VAV3 Genes Are Not Involved with Primary Open-Angle and Primary Angle-Closure Glaucomas in an Indian Population. Investig. Opthalmol. Vis. Sci..

[29] Chowdary P.D., Che D.L., Cui B. (2012). Neurotrophin Signaling via Long-Distance Axonal Transport. Annu. Rev. Phys. Chem..

[30] Chao M.V. (2003). Neurotrophins and their receptors: A convergence point for many signalling pathways. Nat. Rev. Neurosci..

[31] Levkovitch-Verbin H., Bagetta G., Nucci C. (2015). Retinal Ganglion Cell Apoptotic Pathway in Glaucoma. New Trends in Basic and Clinical Research of Glaucoma: A Neurodegenerative Disease of the Visual System, Part A.

[32] Huang E.J., Reichardt L.F. (2001). Neurotrophins: Roles in Neuronal Development and Function. Annu. Rev. Neurosci..

[33] Nafissi N., Foldvari M. (2016). Neuroprotective therapies in glaucoma: I. Neurotrophic factor delivery. Wiley Interdiscip. Rev. Nanomed. Nanobiotechnol..

[34] Skaper S.D. (2012). The neurotrophin family of neurotrophic factors: An overview. Methods Mol. Biol..

[35] Lu B., Pang P.T., Woo N.H. (2005). The yin and yang of neurotrophin action. Nat. Rev. Neurosci..

[36] Drinkut A., Tillack K., Meka D.P., Schulz J.B., Kügler S., Kramer E.R. (2016). Ret is essential to mediate GDNF’s neuroprotective and neuroregenerative effect in a Parkinson disease mouse model. Cell Death Dis..

[37] Huang E.J., Reichardt L.F. (2003). Trk Receptors: Roles in Neuronal Signal Transduction. Annu. Rev. Biochem..

[38] Ito K., Enomoto H. (2016). Retrograde transport of neurotrophic factor signaling: Implications in neuronal development and pathogenesis. J. Biochem..

[39] Oppenheim R. (1991). Cell Death During Development Of The Nervous System. Annu. Rev. Neurosci..

[40] Conradt B. (2009). Genetic Control of Programmed Cell Death During Animal Development. Annu. Rev. Genet..

[41] Chen S.K., Chew K.S., McNeill D.S., Keeley P.W., Ecker J.L., Mao B.Q., Pahlberg J., Kim B., Lee S.C.S., Fox M.A. (2013). Apoptosis Regulates ipRGC Spacing Necessary for Rods and Cones to Drive Circadian Photoentrainment. Neuron.

[42] Oppenheim R.W. (1989). The neurotrophic theory and naturally occurring motoneuron death. Trends Neurosci..

[43] Zweifel L.S., Kuruvilla R., Ginty D.D. (2005). Functions and mechanisms of retrograde neurotrophin signalling. Nat. Rev. Neurosci..

[44] Quigley H.A., Nickells R.W., Kerrigan L.A., Pease M.E., Thibault D.J., Zack D.J. (1995). Retinal ganglion cell death in experimental glaucoma and after axotomy occurs by apoptosis. Invest. Ophthalmol. Vis. Sci..

[45] Yamaguchi Y., Miura M. (2015). Programmed cell death in neurodevelopment. Dev. Cell.

[46] Spalding K.L., Cui Q., Harvey A.R. (1998). The effects of central administration of neurotrophins or transplants of fetal tectal tissue on retinal ganglion cell survival following removal of the superior colliculus in neonatal rats. Dev. Brain Res..

[47] Bear M.F., Connors B.W., Paradiso M.A., Lupash E., Connolly E., Dilernia B., Williams P.C. (2007). The Central Visual System. Neuroscience: Exploring the Brain.

[48] Martersteck E.M., Hirokawa K.E., Evarts M., Bernard A., Duan X., Li Y., Ng L., Oh S.W., Ouellette B., Royall J.J. (2017). Diverse Central Projection Patterns of Retinal Ganglion Cells. Cell Rep..

[49] Whitmore A.V., Libby R.T., John S.W.M. (2005). Glaucoma: Thinking in new ways—a rôle for autonomous axonal self-destruction and other compartmentalised processes?. Prog. Retin. Eye Res..

[50] Harvey A.R., Robertson D. (1992). Time-course and extent of retinal ganglion cell death following ablation of the superior colliculus in neonatal rats. J. Comp. Neurol..

[51] Carpenter P., Sefton A.J., Dreher B., Lim W.-L. (1986). Role of target tissue in regulating the development of retinal ganglion cells in the albino rat: Effects of kainate lesions in the superior colliculus. J. Comp. Neurol..

[52] Perry V.H., Cowey A. (1982). A sensitive period for ganglion cell degeneration and the formation of aberrant retino-fugal connections following tectal lesions in rats. Neuroscience.

[53] Yang X., Chou T.H., Ruggeri M., Porciatti V. (2013). A new mouse model of inducible, chronic retinal ganglion cell dysfunction not associated with cell death. Investig. Ophthalmol. Vis. Sci..

[54] Pearson H.E., Thompson T.P. (1993). Atrophy and Degeneration of Ganglion Cells in Central Retina Following Loss of Postsynaptic Target Neurons in the Dorsal Lateral Geniculate Nucleus of the Adult Cat. Exp. Neurol..

[55] Ellis E.M., Gauvain G., Sivyer B., Murphy G.J. (2016). Shared and distinct retinal input to the mouse superior colliculus and dorsal lateral geniculate nucleus. J. Neurophysiol..

[56] De Groef L., Dekeyster E., Geeraerts E., Lefevere E., Stalmans I., Salinas-Navarro M., Moons L. (2016). Differential visual system organization and susceptibility to experimental models of optic neuropathies in three commonly used mouse strains. Exp. Eye Res..

[57] Chou T.H., Park K.K., Luo X., Porciatti V. (2013). Retrograde signaling in the optic nerve is necessary for electrical responsiveness of retinal ganglion cells. Investig. Ophthalmol. Vis. Sci..

[58] Salinas-Navarro M., Alarcón-Martínez L., Valiente-Soriano F.J., Jiménez-López M., Mayor-Torroglosa S., Avilés-Trigueros M., Villegas-Pérez M.P., Vidal-Sanz M. (2010). Ocular hypertension impairs optic nerve axonal transport leading to progressive retinal ganglion cell degeneration. Exp. Eye Res..

[59] Johansson J.-O. (1988). Inhibition and recovery of retrograde axoplasmic transport in rat optic nerve during and after elevated IOP in vivo. Exp. Eye Res..

[60] Jakobs T.C., Libby R.T., Ben Y., John S.W.M., Masland R.H. (2005). Retinal ganglion cell degeneration is topological but not cell type specific in DBA/2J mice. J. Cell Biol..

[61] Minckler D.S., Bunt A.H., Johanson G.W. (1977). Orthograde and retrograde axoplasmic transport during acute ocular hypertension in the monkey. Invest. Ophthalmol. Vis. Sci..

[62] Valiente-Soriano F.J., Salinas-Navarro M., Jiménez-López M., Alarcón-Martínez L., Ortín-Martínez A., Bernal-Garro J.M., Avilés-Trigueros M., Agudo-Barriuso M., Villegas-Pérez M.P., Vidal-Sanz M. (2015). Effects of Ocular Hypertension in the Visual System of Pigmented Mice. PLoS ONE.

[63] Kim D.H., Kim H.S., Ahn M.D., Chun M.H. (2004). Ganglion Cell Death in Rat Retina by Persistent Intraocular Pressure Elevation. Korean J. Ophthalmol..

[64] Buckingham B.P., Inman D.M., Lambert W., Oglesby E., Calkins D.J., Steele M.R., Vetter M.L., Marsh-Armstrong N., Horner P.J. (2008). Progressive Ganglion Cell Degeneration Precedes Neuronal Loss in a Mouse Model of Glaucoma. J. Neurosci..

[65] Martin K.R.G., Quigley H.A., Valenta D., Kielczewski J., Pease M.E. (2006). Optic nerve dynein motor protein distribution changes with intraocular pressure elevation in a rat model of glaucoma. Exp. Eye Res..

[66] Radius R.L., Anderson D.R. (1981). Rapid axonal transport in primate optic nerve: Distribution of Pressure-Induced Interruption. Arch. Ophthalmol..

[67] Howell G.R., Libby R.T., Jakobs T.C., Smith R.S., Phalan F.C., Barter J.W., Barbay J.M., Marchant J.K., Mahesh N., Porciatti V. (2007). Axons of retinal ganglion cells are insulted in the optic nerve early in DBA/2J glaucoma. J. Cell Biol..

[68] Dengler-Crish C.M., Smith M.A., Inman D.M., Wilson G.N., Young J.W., Crish S.D. (2014). Anterograde transport blockade precedes deficits in retrograde transport in the visual projection of the DBA/2J mouse model of glaucoma. Front. Neurosci..

[69] Quigley H.A., Addicks E.M., Green W.R., Maumenee A.E. (1981). Optic nerve damage in human glaucoma: Ii. The Site of Injury and Susceptibility to Damage. Arch. Ophthalmol..

[70] Anderson D.R., Hendrickson A. (1974). Effect of intraocular pressure on rapid axoplasmic transport in monkey optic nerve. Invest. Ophthalmol..

[71] Crish S.D., Sappington R.M., Inman D.M., Horner P.J., Calkins D.J. (2010). Distal axonopathy with structural persistence in glaucomatous neurodegeneration. Proc. Natl. Acad. Sci. USA.

[72] Crish S.D., Dapper J.D., MacNamee S.E., Balaram P., Sidorova T.N., Lambert W.S., Calkins D.J. (2013). Failure of axonal transport induces a spatially coincident increase in astrocyte BDNF prior to synapse loss in a central target. Neuroscience.

[73] Lambert W.S., Ruiz L., Crish S.D., Wheeler L.A., Calkins D.J. (2011). Brimonidine prevents axonal and somatic degeneration of retinal ganglion cell neurons. Mol. Neurodegener..

[74] Guo Y., Johnson E., Cepurna W., Jia L., Dyck J., Morrison J.C. (2009). Does elevated intraocular pressure reduce retinal TRKB-mediated survival signaling in experimental glaucoma?. Exp. Eye Res..

[75] Scott-Solomon E., Kuruvilla R. (2018). Mechanisms of neurotrophin trafficking via Trk receptors. Mol. Cell. Neurosci..

[76] Schlamp C.L., Li Y., Dietz J.A., Janssen K.T., Nickells R.W. (2006). Progressive ganglion cell loss and optic nerve degeneration in DBA/2J mice is variable and asymmetric. BMC Neurosci..

[77] Agarwal N., Agarwal R., Kumar D.M., Ondricek A., Clark A.F., Wordinger R.J., Pang I.-H. (2007). Comparison of expression profile of neurotrophins and their receptors in primary and transformed rat retinal ganglion cells. Mol. Vis..

[78] Wu Q., Zhang M., Song B.W., Lu B., Hu P. (2007). Expression of ciliary neurotrophic factor after induction of ocular hypertension in the retina of rats. Chin. Med. J. (Engl.).

[79] Kimura A., Namekata K., Guo X., Harada C., Harada T. (2016). Neuroprotection, growth factors and BDNF-TRKB signalling in retinal degeneration. Int. J. Mol. Sci..

[80] Beltran W.A., Zhang Q., Kijas J.W., Gu D., Rohrer H., Jordan J.A., Aguirre G.D. (2003). Cloning, mapping, and retinal expression of the canine ciliary neurotrophic factor receptor α (CNTFRα). Investig. Ophthalmol. Vis. Sci..

[81] Koeberle P.D., Ball A.K. (2002). Neurturin enhances the survival of axotomized retinal ganglion cells in vivo: Combined effects with glial cell line-derived neurotrophic factor and brain-derived neurotrophic factor. Neuroscience.

[82] Miotke J.A., MacLennan A.J., Meyer R.L. (2007). Immunohistochemical localization of CNTFRα in adult mouse retina and optic nerve following intraorbital nerve crush: Evidence for the axonal loss of a trophic factor receptor after injury. J. Comp. Neurol..

[83] Parmhans N., Sajgo S., Niu J., Luo W., Badea T.C. (2018). Characterization of retinal ganglion cell, horizontal cell, and amacrine cell types expressing the neurotrophic receptor tyrosine kinase Ret. J. Comp. Neurol..

[84] Hofer M., Pagliusi S.R., Hohn A., Leibrock J., Barde Y.A. (1990). Regional distribution of brain-derived neurotrophic factor mRNA in the adult mouse brain. EMBO J..

[85] Wetmore C., Ernfors P., Persson H., Olson L. (1990). Localization of brain-derived neurotrophic factor mRNA to neurons in the brain by in situ hybridization. Exp. Neurol..

[86] Dekeyster E., Geeraerts E., Buyens T., Van Den Haute C., Baekelandt V., De Groef L., Salinas-Navarro M., Moons L. (2015). Tackling glaucoma from within the brain: An unfortunate interplay of BDNF and TrkB. PLoS ONE.

[87] Tanaka H., Ito Y., Nakamura S., Shimazawa M., Hara H. (2009). Involvement of brain-derived neurotrophic factor in time-dependent neurodegeneration in the murine superior colliculus after intravitreal injection of N-methyl-D-aspartate. Mol. Vis..

[88] Ito Y., Shimazawa M., Inokuchi Y., Fukumitsu H., Furukawa S., Araie M., Hara H. (2008). Degenerative alterations in the visual pathway after NMDA-induced retinal damage in mice. Brain Res..

[89] Pöyhönen S., Er S., Domanskyi A., Airavaara M. (2019). Effects of Neurotrophic Factors in Glial Cells in the Central Nervous System: Expression and Properties in Neurodegeneration and Injury. Front. Physiol..

[90] Pease M.E., McKinnon S.J., Quigley H.A., Kerrigan-Baumrind L.A., Zack D.J. (2000). Obstructed axonal transport of BDNF and its receptor TrkB in experimental glaucoma. Invest. Ophthalmol. Vis. Sci..

[91] Quigley H.A., McKinnon S.J., Zack D.J., Pease M.E., Kerrigan-Baumrind L.A., Kerrigan D.F., Mitchell R.S. (2000). Retrograde axonal transport of BDNF in retinal ganglion cells is blocked by acute IOP elevation in rats. Invest. Ophthalmol. Vis. Sci..

[92] Yan Q., Wang J., Matheson C.R., Urich J.L. (1999). Glial cell line-derived neurotrophic factor (GDNF) promotes the survival of axotomized retinal ganglion cells in adult rats: Comparison to and combination with brain-derived neurotrophic factor (BDNF). J. Neurobiol..

[93] Takihara Y., Inatani M., Hayashi H., Adachi N., Iwao K., Inoue T., Iwao M., Tanihara H. (2011). Dynamic imaging of axonal transport in living retinal ganglion cells in vitro. Investig. Ophthalmol. Vis. Sci..

[94] Ghaffariyeh A., Honarpisheh N., Shakiba Y., Puyan S., Chamacham T., Zahedi F., Zarrineghbal M. (2009). Brain-derived neurotrophic factor in patients with normal-tension glaucoma. Optom. J. Am. Optom. Assoc..

[95] Ghaffariyeh A., Honarpisheh N., Heidari M.H., Puyan S., Abasov F. (2011). Brain-Derived Neurotrophic Factor as a Biomarker in Primary Open-Angle Glaucoma. Optom. Vis. Sci..

[96] Shpak A.A., Guekht A.B., Druzhkova T.A., Kozlova K.I., Gulyaeva N.V. (2018). Brain-Derived Neurotrophic Factor in Patients with Primary Open-Angle Glaucoma and Age-related Cataract. Curr. Eye Res..

[97] Pietrucha-Dutczak M., Amadio M., Govoni S., Lewin-Kowalik J., Smedowski A. (2018). The role of endogenous neuroprotective mechanisms in the prevention of retinal ganglion cells degeneration. Front. Neurosci..

[98] Perez M.-T.R., Caminos E. (1995). Expression of brain-derived neurotrophic factor and of its functional receptor in neonatal and adult rat retina. Neurosci. Lett..

[99] Lambert W., Agarwal R., Howe W., Clark A.F., Wordinger R.J. (2001). Neurotrophin and neurotrophin receptor expression by cells of the human lamina cribrosa. Invest. Ophthalmol. Vis. Sci..

[100] Vecino E., Garía-Grespo D., Garía M., Martinez-Millán L., Sharma S.C., Carrascal E. (2002). Rat retinal ganglion cells co-express brain derived neurotrophic factor (BDNF) and its receptor TrkB. Vis. Res..

[101] Johnson E.C., Deppmeier L.M.H., Wentzien S.K.F., Hsu I., Morrison J.C. (2000). Chronology of optic nerve head and retinal responses to elevated intraocular pressure. Investig. Ophthalmol. Vis. Sci..

[102] Herzog K.-H., von Bartheld C.S. (1998). Contributions of the Optic Tectum and the Retina as Sources of Brain-Derived Neurotrophic Factor for Retinal Ganglion Cells in the Chick Embryo. J. Neurosci..

[103] Seki M., Tanaka T., Sakai Y., Fukuchi T., Abe H., Nawa H., Takei N. (2005). Müller Cells as a Source of Brain-derived Neurotrophic Factor in the Retina: Noradrenaline Upregulates Brain-derived Neurotrophic Factor Levels in Cultured Rat Müller Cells. Neurochem. Res..

[104] García M., Forster V., Hicks D., Vecino E. (2003). In vivo expression of neurotrophins and neurotrophin receptors is conserved in adult porcine retina in vitro. Investig. Ophthalmol. Vis. Sci..

[105] Chong R.S., Martin K.R. (2015). Glial cell interactions and glaucoma. Curr. Opin. Ophthalmol..

[106] Galindo-Romero C., Valiente-Soriano F.J., Jiménez-López M., García-Ayuso D., Villegas-Pérez M.P., Vidal-Sanz M., Agudo-Barriuso M. (2013). Effect of brain-derived neurotrophic factor on mouse axotomized retinal ganglion cells and phagocytic microglia. Investig. Ophthalmol. Vis. Sci..

[107] Harada T., Harada C., Kohsaka S., Wada E., Yoshida K., Ohno S., Mamada H., Tanaka K., Parada L.F., Wada K. (2002). Microglia-Müller glia cell interactions control neurotrophic factor production during light-induced retinal degeneration. J. Neurosci..

[108] Langmann T. (2007). Microglia activation in retinal degeneration. J. Leukoc. Biol..

[109] Inanc Tekin M., Sekeroglu M.A., Demirtas C., Tekin K., Doguizi S., Bayraktar S., Yilmazbas P. (2018). Brain-Derived Neurotrophic Factor in Patients With Age-Related Macular Degeneration and Its Correlation With Retinal Layer Thicknesses. Investig. Opthalmol. Vis. Sci..

[110] Chitranshi N., Dheer Y., Abbasi M., You Y., Graham S.L., Gupta V. (2018). Glaucoma Pathogenesis and Neurotrophins: Focus on the Molecular and Genetic Basis for Therapeutic Prospects. Curr. Neuropharmacol..

[111] Di Polo A., Aigner L.J., Dunn R.J., Bray G.M., Aguayo A.J. (1998). Prolonged delivery of brain-derived neurotrophic factor by adenovirus-infected Muller cells temporarily rescues injured retinal ganglion cells. Proc. Natl. Acad. Sci. USA.

[112] Clarke D.B., Bray G.M., Aguayo A.J. (1998). Prolonged administration of NT-4/5 fails to rescue most axotomized retinal ganglion cells in adult rats. Vis. Res..

[113] Frank L., Ventimiglia R., Anderson K., Lindsay R.M., Rudge J.S. (1996). BDN F Down-regulates Neurotrophin Responsiveness, TrkB Protein and TrkB mRNA Levels in Cultured Rat Hippocampal Neurons. Eur. J. Neurosci..

[114] Chen H., Weber A.J. (2004). Brain-derived neurotrophic factor reduces TrkB protein and mRNA in the normal retina and following optic nerve crush in adult rats. Brain Res..

[115] Ratican S.E., Osborne A., Martin K.R. (2018). Progress in Gene Therapy to Prevent Retinal Ganglion Cell Loss in Glaucoma and Leber’s Hereditary Optic Neuropathy. Neural Plast..

[116] Proenca C.C., Song M., Lee F.S. (2016). Differential effects of BDNF and neurotrophin 4 (NT4) on endocytic sorting of TrkB receptors. J. Neurochem..

[117] Osborne A., Wang A.X.Z., Tassoni A., Widdowson P.S., Martin K.R. (2018). Design of a Novel Gene Therapy Construct to Achieve Sustained Brain-Derived Neurotrophic Factor Signaling in Neurons. Hum. Gene Ther..

[118] Cheng L., Sapieha P., Kittlerova P., Hauswirth W.W., Di Polo A. (2002). TrkB gene transfer protects retinal ganglion cells from axotomy-induced death in vivo. J. Neurosci..

[119] Nuzzi R., Tridico F. (2017). Glaucoma: Biological trabecular and neuroretinal pathology with perspectives of therapy innovation and preventive diagnosis. Front. Neurosci..

[120] Johnson T.V., Bull N.D., Martin K.R. (2011). Neurotrophic factor delivery as a protective treatment for glaucoma. Exp. Eye Res..

[121] Zhou Y., Pernet V., Hauswirth W.W., Di Polo A. (2005). Activation of the Extracellular Signal-Regulated Kinase 1/2 Pathway by AAV Gene Transfer Protects Retinal Ganglion Cells in Glaucoma. Mol. Ther..

[122] Malik J.M.I., Shevtsova Z., Bähr M., Kügler S. (2005). Long-term in vivo inhibition of CNS neurodegeneration by Bcl-XL gene transfer. Mol. Ther..

[123] Planchamp V., Bermel C., Tönges L., Ostendorf T., Kügler S., Reed J.C., Kermer P., Bähr M., Lingor P. (2008). BAG1 promotes axonal outgrowth and regeneration in vivo via Raf-1 and reduction of ROCK activity. Brain.

[124] McKinnon S.J., Lehman D.M., Tahzib N.G., Ransom N.L., Reitsamer H.A., Liston P., LaCasse E., Li Q., Korneluk R.G., Hauswirth W.W. (2002). Baculoviral IAP repeat-containing-4 protects optic nerve axons in a rat glaucoma model. Mol. Ther..

[125] Kügler S., Klöcker N., Kermer P., Isenmann S., Bähr M. (1999). Transduction of axotomized retinal ganglion cells by adenoviral vector administration at the optic nerve stump: An in vivo model system for the inhibition of neuronal apoptotic cell death. Gene Ther..

[126] Kermer P., Klöcker N., Labes M., Bähr M. (1998). Inhibition of CPP32-Like Proteases Rescues Axotomized Retinal Ganglion Cells from Secondary Cell Death In Vivo. J. Neurosci..

[127] Thoenen H., Sendtner M. (2002). Neurotrophins: From enthusiastic expectations through sobering experiences to rational therapeutic approaches. Nat. Neurosci..

[128] Barker P.A. (1998). p75NTR: A study in contrasts. Cell Death Differ..

[129] Bai Y., Xu J., Brahimi F., Zhuo Y., Sarunic M.V., Uri Saragovi H. (2010). An agonistic TrKb mAb causes sustained TrkB activation, delays RGC death, and protects the retinal structure in optic nerve axotomy and in glaucoma. Investig. Ophthalmol. Vis. Sci..

[130] Bai Y., Dergham P., Nedev H., Xu J., Galan A., Rivera J.C., ZhiHua S., Mehta H.M., Woo S.B., Sarunic M.V. (2010). Chronic and Acute Models of Retinal Neurodegeneration TrkA Activity Are Neuroprotective whereas p75 NTR Activity Is Neurotoxic through a Paracrine Mechanism. J. Biol. Chem..

[131] Gupta V.K., You Y., Li J.C., Klistorner A., Graham S.L. (2013). Protective Effects of 7,8-Dihydroxyflavone on Retinal Ganglion and RGC-5 Cells Against Excitotoxic and Oxidative Stress. J. Mol. Neurosci..

[132] Rheaume B.A., Jereen A., Bolisetty M., Sajid M.S., Yang Y., Renna K., Sun L., Robson P., Trakhtenberg E.F. (2018). Single cell transcriptome profiling of retinal ganglion cells identifies cellular subtypes. Nat. Commun..

[133] Logan A., Ahmed Z., Baird A., Gonzalez A.M., Berry M. (2006). Neurotrophic factor synergy is required for neuronal survival and disinhibited axon regeneration after CNS injury. Brain.

[134] Flachsbarth K., Jankowiak W., Kruszewski K., Helbing S., Bartsch S., Bartsch U. (2018). Pronounced synergistic neuroprotective effect of GDNF and CNTF on axotomized retinal ganglion cells in the adult mouse. Exp. Eye Res..

[135] Kitamura Y., Bikbova G., Baba T., Yamamoto S., Oshitari T. (2019). In vivo effects of single or combined topical neuroprotective and regenerative agents on degeneration of retinal ganglion cells in rat optic nerve crush model. Sci. Rep..

[136] Pease M.E., Zack D.J., Berlinicke C., Bloom K., Cone F., Wang Y., Klein R.L., Hauswirth W.W., Quigley H.A. (2009). Effect of cntf on retinal ganglion cell survival in experimental glaucoma. Investig. Ophthalmol. Vis. Sci..

[137] Klöcker N., Kermer P., Weishaupt J.H., Labes M., Ankerhold R., Bähr M. (2000). Brain-derived neurotrophic factor-mediated neuroprotection of adult rat retinal ganglion cells in vivo does not exclusively depend on phosphatidyl-inositol-3′-kinase/protein kinase B signaling. J. Neurosci..

[138] West A.E., Pruunsild P., Timmusk T. (2014). Neurotrophins: Transcription and translation. Handb. Exp. Pharmacol..

[139] Corredor R.G., Goldberg J.L. (2009). Electrical activity enhances neuronal survival and regeneration. J. Neural Eng..

[140] Kolarow R., Kuhlmann C.R.W., Munsch T., Zehendner C., Brigadski T., Luhmann H.J., Lessmann V. (2014). BDNF-induced nitric oxide signals in cultured rat hippocampal neurons: Time course, mechanism of generation, and effect on neurotrophin secretion. Front. Cell. Neurosci..

[141] Sehic A., Guo S., Cho K.-S., Corraya R.M., Chen D.F., Utheim T.P. (2016). Electrical Stimulation as a Means for Improving Vision. Am. J. Pathol..

[142] Manthey A.L., Liu W., Jiang Z.X., Lee M.H.K., Ji J., So K.-F., Lai J.S.M., Lee V.W.H., Chiu K. (2017). Using Electrical Stimulation to Enhance the Efficacy of Cell Transplantation Therapies for Neurodegenerative Retinal Diseases: Concepts, Challenges, and Future Perspectives. Cell Transplant..

[143] Sato T., Fujikado T., Lee T.-S., Tano Y. (2008). Direct Effect of Electrical Stimulation on Induction of Brain-Derived Neurotrophic Factor from Cultured Retinal Müller Cells. Investig. Opthalmol. Vis. Sci..

[144] Zhou W.T., Ni Y.Q., Jin Z.B., Zhang M., Wu J.H., Zhu Y., Xu G.Z., Gan D.K. (2012). Electrical stimulation ameliorates light-induced photoreceptor degeneration in vitro via suppressing the proinflammatory effect of microglia and enhancing the neurotrophic potential of Müller cells. Exp. Neurol..

[145] Sato T., Fujikado T., Morimoto T., Matsushita K., Harada T., Tano Y. (2008). Effect of electrical stimulation on IGF-1 transcription by L-type calcium channels in cultured retinal Müller cells. Jpn. J. Ophthalmol..

[146] Ni Y., Gan D., Xu H., Xu G., Da C. (2009). Neuroprotective effect of transcorneal electrical stimulation on light-induced photoreceptor degeneration. Exp. Neurol..

[147] Ciavatta V.T., Kim M., Wong P., Nickerson J.M., Shuler R.K., Mclean G.Y., Pardue M.T. (2009). Retinal expression of Fgf2 in RCS rats with subretinal microphotodiode array. Investig. Ophthalmol. Vis. Sci..

[148] Morimoto T., Miyoshi T., Matsuda S., Tano Y., Fujikado T., Fukuda Y. (2005). Transcorneal electrical stimulation rescues axotomized retinal ganglion cells by activating endogenous retinal IGF-1 system. Investig. Ophthalmol. Vis. Sci..

[149] Willmann G., Schäferhoff K., Fischer M.D., Arango-Gonzalez B., Bolz S., Naycheva L., Röck T., Bonin M., Bartz-Schmidt K.U., Zrenner E. (2011). Gene expression profiling of the retina after transcorneal electrical stimulation in wild-type Brown Norway rats. Investig. Ophthalmol. Vis. Sci..

[150] Henrich-Noack P., Lazik S., Sergeeva E., Wagner S., Voigt N., Prilloff S., Fedorov A., Sabel B.A. (2013). Transcorneal alternating current stimulation after severe axon damage in rats results in “long-term silent survivor” neurons. Brain Res. Bull..

[151] Osako T., Chuman H., Maekubo T., Ishiai M., Kawano N., Nao-i N. (2013). Effects of steroid administration and transcorneal electrical stimulation on the anatomic and electrophysiologic deterioration of nonarteritic ischemic optic neuropathy in a rodent model. Jpn. J. Ophthalmol..

[152] Fujikado T., Morimoto T., Matsushita K., Shimojo H., Okawa Y., Tano Y. (2006). Effect of Transcorneal Electrical Stimulation in Patients with Nonarteritic Ischemic Optic Neuropathy or Traumatic Optic Neuropathy. Jpn. J. Ophthalmol..

[153] Gall C., Fedorov A.B., Ernst L., Borrmann A., Sabel B.A. (2010). Repetitive transorbital alternating current stimulation in optic neuropathy. NeuroRehabilitation.

[154] Gall C., Sgorzaly S., Schmidt S., Brandt S., Fedorov A., Sabel B.A. (2011). Noninvasive transorbital alternating current stimulation improves subjective visual functioning and vision-related quality of life in optic neuropathy. Brain Stimul..

[155] Fedorov A., Jobke S., Bersnev V., Chibisova A., Chibisova Y., Gall C., Sabel B.A. (2011). Restoration of vision after optic nerve lesions with noninvasive transorbital alternating current stimulation: A clinical observational study. Brain Stimul..

[156] Gall C., Schmidt S., Schittkowski M.P., Antal A., Ambrus G.G., Paulus W., Dannhauer M., Michalik R., Mante A., Bola M. (2016). Alternating current stimulation for vision restoration after optic nerve damage: A randomized clinical trial. PLoS ONE.

[157] Weber A.J., Viswanáthan S., Ramanathan C., Harman C.D. (2010). Combined application of BDNF to the eye and brain enhances ganglion cell survival and function in the cat after optic nerve injury. Investig. Ophthalmol. Vis. Sci..

[158] Segal R.A. (2003). Selectivity in neurotrophin signaling: Theme and variations. Annu. Rev. Neurosci..

[159] Quigley H.A., Addicks E.M. (1980). Chronic experimental glaucoma in primates. II. Effect of extended intraocular pressure elevation on optic nerve head and axonal transport. Invest. Ophthalmol. Vis. Sci..

[160] Cosker K.E., Courchesne S.L., Segal R.A. (2008). Action in the axon: Generation and transport of signaling endosomes. Curr. Opin. Neurobiol..

[161] Howe C.L., Valletta J.S., Rusnak A.S., Mobley W.C. (2001). NGF Signaling from Clathrin-Coated Vesicles. Neuron.

[162] Delcroix J.-D., Valletta J.S., Wu C., Hunt S.J., Kowal A.S., Mobley W.C. (2003). NGF Signaling in Sensory Neurons. Neuron.

[163] Zheng J., Shen W.H., Lu T.J., Zhou Y., Chen Q., Wang Z., Xiang T., Zhu Y.C., Zhang C., Duan S. (2008). Clathrin-dependent endocytosis is required for TrkB-dependent Akt-mediated neuronal protection and dendritic growth. J. Biol. Chem..

[164] Qu J., Wang D., Grosskreutz C.L. (2010). Mechanisms of retinal ganglion cell injury and defense in glaucoma. Exp. Eye Res..

[165] Ibáñez C.F. (2007). Message in a bottle: Long-range retrograde signaling in the nervous system. Trends Cell Biol..

[166] Harrington A.W., Ginty D.D. (2013). Long-distance retrograde neurotrophic factor signalling in neurons. Nat. Rev. Neurosci..

[167] Ye M., Lehigh K.M., Ginty D.D. (2018). Multivesicular bodies mediate long-range retrograde NGF-TrkA signaling. Elife.

[168] Cosker K.E., Segal R.A. (2014). Neuronal signaling through endocytosis. Cold Spring Harb. Perspect. Biol..

[169] Lom B., Cogen J., Sanchez A.L., Vu T., Cohen-Cory S. (2018). Local and Target-Derived Brain-Derived Neurotrophic Factor Exert Opposing Effects on the Dendritic Arborization of Retinal Ganglion Cells In Vivo. J. Neurosci..

[170] Watson F.L., Heerssen H.M., Bhattacharyya A., Klesse L., Lin M.Z., Segal R.A. (2001). Neurotrophins use the Erk5 pathway to mediate a retrograde survival response. Nat. Neurosci..

[171] Van Oterendorp C., Sgouris S., Schallner N., Biermann J., Lagrèze W.A. (2014). Retrograde neurotrophic signaling in rat retinal ganglion cells is transmitted via the ERK5 but not the ERK1/2 pathway. Investig. Ophthalmol. Vis. Sci..

[172] Geeraerts E., Claes M., Dekeyster E., Salinas-Navarro M., De Groef L., Van den Haute C., Scheyltjens I., Baekelandt V., Arckens L., Moons L. (2019). Optogenetic Stimulation of the Superior Colliculus Confers Retinal Neuroprotection in a Mouse Glaucoma Model. J. Neurosci..

[173] Hasel P., Dando O., Jiwaji Z., Baxter P., Todd A.C., Heron S., Márkus N.M., McQueen J., Hampton D.W., Torvell M. (2017). Neurons and neuronal activity control gene expression in astrocytes to regulate their development and metabolism. Nat. Commun..

[174] Galindo-Romero C., Avilés-Trigueros M., Jiménez-López M., Valiente-Soriano F.J., Salinas-Navarro M., Nadal-Nicolás F., Villegas-Pérez M.P., Vidal-Sanz M., Agudo-Barriuso M. (2011). Axotomy-induced retinal ganglion cell death in adult mice: Quantitative and topographic time course analyses. Exp. Eye Res..

[175] Heiduschka P., Thanos S. (2000). Restoration of the retinofugal pathway. Prog. Retin. Eye Res..

[176] (1999). Intrinsic survival mechanisms for retinal ganglion cells. Eur. J. Ophthalmol..

[177] Humphrey M.F., Beazley L.D. (1985). Retinal ganglion cell death during optic nerve regeneration in the froghyla moorei. J. Comp. Neurol..

[178] Beazley L.D., Darbyy J.E., Perry V.H. (1986). Cell death in the retinal ganglion cell layer during optic nerve regeneration for the frog Rana pipiens. Vis. Res..

[179] WATANABE M., INUKAI N., FUKUDA Y. (2001). Survival of retinal ganglion cells after transection of the optic nerve in adult cats: A quantitative study within two weeks. Vis. Neurosci..

[180] Muchnick N., Hibbard E. (1980). Avian retinal ganglion cells resistant to degeneration after optic nerve lesion. Exp. Neurol..

[181] Soto I., Marie B., Baro D.J., Blanco R.E. (2003). FGF-2 modulates expression and distribution of GAP-43 in frog retinal ganglion cells after optic nerve injury. J. Neurosci. Res..

[182] Duprey-Díaz M.V., Blagburn J.M., Blanco R.E. (2016). Exogenous Modulation of Retinoic Acid Signaling Affects Adult RGC Survival in the Frog Visual System after Optic Nerve Injury. PLoS ONE.

[183] Bollaerts I., Veys L., Geeraerts E., Andries L., De Groef L., Buyens T., Salinas-Navarro M., Moons L., Van Hove I. (2018). Complementary research models and methods to study axonal regeneration in the vertebrate retinofugal system. Brain Struct. Funct..

[184] Scalia F., Arango V., Singman E.L. (1985). Loss and displacement of ganglion cells after optic nerve regeneration in adultRana pipiens. Brain Res..

[185] Williams D.L. (2017). Regenerating reptile retinas: A comparative approach to restoring retinal ganglion cell function. Eye.

[186] Ramón Y., Cajal S., DeFelipe J., Jones E.G., May R.M., DeFelipe J., Jones E.G. (2012). Cajal’s Degeneration and Regeneration of the Nervous System.

[187] Germain F., Calvo M., de la Villa P. (2004). Rabbit retinal ganglion cell survival after optic nerve section and its effect on the inner plexiform layer. Exp. Eye Res..

[188] Berkelaar M., Clarke D., Wang Y., Bray G., Aguayo A. (1994). Axotomy results in delayed death and apoptosis of retinal ganglion cells in adult rats. J. Neurosci..

[189] Magharious M.M., D’Onofrio P.M., Koeberle P.D. (2011). Optic Nerve Transection: A Model of Adult Neuron Apoptosis in the Central Nervous System. J. Vis. Exp..

[190] Grafstein B., Ingoglia N.A. (1982). Intracranial transection of the optic nerve in adult mice: Preliminary observations. Exp. Neurol..

[191] Allcutt D., Berry M., Sievers J. (1984). A qualitative comparison of the reactions of retinal ganglion cell axons to optic nerve crush in neonatal and adult mice. Dev. Brain Res..

[192] Misantone L.J., Gershenbaum M., Murray M. (1984). Viability of retinal ganglion cells after optic nerve crush in adult rats. J. Neurocytol..

[193] Agudo M., Pérez-Marín M.C., Lönngren U., Sobrado P., Conesa A., Cánovas I., Salinas-Navarro M., Miralles-Imperial J., Hallböök F., Vidal-Sanz M. (2008). Time course profiling of the retinal transcriptome after optic nerve transection and optic nerve crush. Mol. Vis..

[194] Li H.-J., Sun Z.-L., Yang X.-T., Zhu L., Feng D.-F. (2017). Exploring Optic Nerve Axon Regeneration. Curr. Neuropharmacol..

[195] Villegas-Pérez M.-P., Vidal-Sanz M., Rasminsky M., Bray G.M., Aguayo A.J. (1993). Rapid and protracted phases of retinal ganglion cell loss follow axotomy in the optic nerve of adult rats. J. Neurobiol..

[196] Peinado-Ramón P., Salvador M., Villegas-Pérez M.P., Vidal-Sanz M. (1996). Effects of axotomy and intraocular administration of NT-4, NT-3, and brain-derived neurotrophic factor on the survival of adult rat retinal ganglion cells. A quantitative in vivo study. Invest. Ophthalmol. Vis. Sci..

[197] Bähr M. (2000). Live or let die—Retinal ganglion cell death and survival during development and in the lesioned adult CNS. Trends Neurosci..

[198] Isenmann S., Wahl C., Krajewski S., Reed J.C., Bähr M. (1997). Up-regulation of Bax protein in degenerating retinal ganglion cells precedes apoptotic cell death after optic nerve lesion in the rat. Eur. J. Neurosci..

[199] Leaver S.G., Cui Q., Plant G.W., Arulpragasam A., Hisheh S., Verhaagen J., Harvey A.R. (2006). AAV-mediated expression of CNTF promotes long-term survival and regeneration of adult rat retinal ganglion cells. Gene Ther..

[200] Murray M., Grafstein B. (1969). Changes in the morphology and amino acid incorporation of regenerating goldfish optic neurons. Exp. Neurol..

[201] Kato S., Matsukawa T., Koriyama Y., Sugitani K., Ogai K. (2013). A molecular mechanism of optic nerve regeneration in fish: The retinoid signaling pathway. Prog. Retin. Eye Res..

[202] Zou S., Tian C., Ge S., Hu B. (2013). Neurogenesis of Retinal Ganglion Cells Is Not Essential to Visual Functional Recovery after Optic Nerve Injury in Adult Zebrafish. PLoS ONE.

[203] Zou S., Yin W., Huang Y., Tian C., Ge S., Hu B. (2015). Functional Regeneration and Remyelination in the Zebrafish Optic Nerve. Neural Regeneration.

[204] Rodger J., Dunlop S.A. (2015). Central Nerve Regeneration in Reptiles. Neural Regeneration.

[205] Beckers A., Van Dyck A., Bollaerts I., Van houcke J., Lefevere E., Andries L., Agostinone J., Van Hove I., Di Polo A., Lemmens K. (2019). An Antagonistic Axon-Dendrite Interplay Enables Efficient Neuronal Repair in the Adult Zebrafish Central Nervous System. Mol. Neurobiol..

[206] Wyatt C., Ebert A., Reimer M.M., Rasband K., Hardy M., Chien C.-B., Becker T., Becker C.G. (2010). Analysis of the astray/robo2 Zebrafish Mutant Reveals that Degenerating Tracts Do Not Provide Strong Guidance Cues for Regenerating Optic Axons. J. Neurosci..

[207] McCurley A.T., Callard G.V.G.V. (2010). Time course analysis of gene expression patterns in zebrafish eye during optic nerve regeneration. J. Exp. Neurosci..

[208] Dunlop S.A., Tennant M., Beazley L.D. (2002). Extent of retinal ganglion cell death in the frogLitoria moorei after optic nerve regeneration induced by lesions of different sizes. J. Comp. Neurol..

[209] Zhao Y., Szaro B.G. (1994). The return of phosphorylated and nonphosphorylated epitopes of neurofilament proteins to the regenerating optic nerve ofXenopus laevis. J. Comp. Neurol..

[210] Liu Y., Yu H., Deaton S.K., Szaro B.G. (2012). Heterogeneous Nuclear Ribonucleoprotein K, an RNA-Binding Protein, Is Required for Optic Axon Regeneration in Xenopus laevis. J. Neurosci..

[211] Stelzner D.J., Bohn R.C., Strauss J.A. (1986). Regeneration of the frog optic nerve. Neurochem. Pathol..

[212] Dunlop S.A. (2003). Axonal sprouting in the optic nerve is not a prerequisite for successful regeneration. J. Comp. Neurol..

[213] Matsukawa T., Arai K., Koriyama Y., Liu Z., Kato S. (2004). Axonal Regeneration of Fish Optic Nerve after Injury. Biol. Pharm. Bull..

[214] Koriyama Y., Homma K., Sugitani K., Higuchi Y., Matsukawa T., Murayama D., Kato S. (2007). Upregulation of IGF-I in the goldfish retinal ganglion cells during the early stage of optic nerve regeneration. Neurochem. Int..

[215] Homma K., Koriyama Y., Mawatari K., Higuchi Y., Kosaka J., Kato S. (2007). Early downregulation of IGF-I decides the fate of rat retinal ganglion cells after optic nerve injury. Neurochem. Int..

[216] Lenkowski J.R., Raymond P.A. (2014). Müller glia: Stem cells for generation and regeneration of retinal neurons in teleost fish. Prog. Retin. Eye Res..

[217] Cudeiro J., Rivadulla C. (1999). Sight and insight – on the physiological role of nitric oxide in the visual system. Trends Neurosci..

[218] Koriyama Y., Yasuda R., Homma K., Mawatari K., Nagashima M., Sugitani K., Matsukawa T., Kato S. (2009). Nitric oxide-cGMP signaling regulates axonal elongation during optic nerve regeneration in the goldfish in vitro and in vivo. J. Neurochem..

[219] Lee E.J., Kim K.Y., Gu T.H., Moon J.I., Kim I.B., Lee M.Y., Oh S.J., Chun M.H. (2003). Neuronal nitric oxide synthase is expressed in the axotomized ganglion cells of the rat retina. Brain Res..

[220] Koeberle P.D., Ball A.K. (1999). Nitric Oxide Synthase Inhibition Delays Axonal Degeneration and Promotes the Survival of Axotomized Retinal Ganglion Cells. Exp. Neurol..

[221] Canossa M., Giordano E., Cappello S., Guarnieri C., Ferri S. (2002). Nitric oxide down-regulates brain-derived neurotrophic factor secretion in cultured hippocampal neurons. Proc. Natl. Acad. Sci. USA.

[222] Chidlow G., Wood J.P.M., Casson R.J. (2014). Expression of inducible heat shock proteins Hsp27 and Hsp70 in the visual pathway of rats subjected to various models of retinal ganglion cell injury. PLoS ONE.

[223] Kamioka Y., Fujikawa C., Ogai K., Sugitani K., Watanabe S., Kato S., Wakasugi K. (2013). Functional characterization of fish neuroglobin: Zebrafish neuroglobin is highly expressed in amacrine cells after optic nerve injury and can translocate into ZF4 cells. Biochim. Biophys. Acta Proteins Proteom..

[224] Sugitani K., Koriyama Y., Ogai K., Wakasugi K., Kato S. (2016). A Possible Role of Neuroglobin in the Retina After Optic Nerve Injury: A Comparative Study of Zebrafish and Mouse Retina. Adv. Exp. Med. Biol..

[225] Rosenzweig S., Raz-Prag D., Nitzan A., Galron R., Paz M., Jeserich G., Neufeld G., Barzilai A., Solomon A.S. (2010). Sema-3A indirectly disrupts the regeneration process of goldfish optic nerve after controlled injury. Graefes Arch. Clin. Exp. Ophthalmol..

[226] Shirvan A., Kimron M., Holdengreber V., Ziv I., Ben-Shaul Y., Melamed S., Melamed E., Barzilai A., Solomon A.S. (2002). Anti-semaphorin 3A Antibodies Rescue Retinal Ganglion Cells from Cell Death following Optic Nerve Axotomy. J. Biol. Chem..

[227] Ogai K., Nishitani M., Kuwana A., Mawatari K., Koriyama Y., Sugitani K., Nakashima H., Kato S. (2014). Regeneration-associated genes on optic nerve regeneration in fish retina. Adv. Exp. Med. Biol..

